# Modifiers of effectiveness and deterioration, and prognostic factors of adherence in internet-based interventions for treatment of perinatal depression: A systematic review and individual participant data meta-analysis

**DOI:** 10.1016/j.invent.2026.100993

**Published:** 2026-09-03

**Authors:** Marketa Ciharova, Pim Cuijpers, Mathias Harrer, Yafit Hirshler, Olga-Maria Panagiotopoulou, Lingyao Tong, Clara Miguel, Iryna Frankova, Alan W. Gemmill, Claudia Buntrock, Wouter van Ballegooijen, Ana Fonseca, Mariana Branquinho, Erik Forsell, Nicole E. Pugh, Heather D. Hadjistavropoulos, Yaoyao Sun, Pamela Franco, Carlos Barquero-Jiménez, Irene Gómez-Gómez, Emma Motrico, Pedro Fonseca Zuccolo, Daniel Fatori, Jeannette Milgrom, Eirini Karyotaki

**Affiliations:** a Department of Clinical, Neuro & Developmental Psychology, Amsterdam Public Health Research Institute, Vrije Universiteit Amsterdam, Amsterdam, the Netherlands; b WHO Collaborating Centre for Research and Dissemination of Psychological Interventions, Vrije Universiteit Amsterdam, the Netherlands; c Babeș-Bolyai University, International Institute for Psychotherapy, Cluj-Napoca, Romania; d Department of Psychiatry and Psychotherapy, School of Medicine and Health, Technical University of Munich, Munich, Germany; e Parent-Infant Research Institute, Heidelberg Repatriation Hospital, Heidelberg Heights, Australia; f Department of Clinical Psychology, University of Amsterdam, Amsterdam, the Netherlands; g Center for Research in Epidemiology and Statistics (CRESS), Paris, France; h Department of Medical Psychology, Psychosomatic Medicine and Psychotherapy, Bogomolets National Medical University, Kyiv, Ukraine; i ARQ National Psychotrauma Centre/ARQ Centrum 45, Diemen/Oegstgeest, Oegstgeest, Netherlands; j Institute of Social Medicine and Health Systems Research, Medical Faculty, Otto-von-Guericke-University Magdeburg, Magdeburg, Germany; k Department of Psychiatry, Amsterdam University Medical Center, Amsterdam, the Netherlands; l University of Coimbra, Center for Research in Neuropsychology and Cognitive-Behavioral Intervention, Coimbra, Portugal; m Centre for Psychiatry Research, Department of Clinical Neuroscience, Karolinska Institutet, Sweden; n Department of Psychology, University of Regina, Regina, SK, Canada; o Peking University Sixth Hospital, Peking University Institute of Mental Health, NHC Key Laboratory of Mental Health (Peking University), National Clinical Research Center for Mental Disorders (Peking University Sixth Hospital), Beijing, China; p School of Psychology, Pontificia Universidad Católica de Chile, Santiago, Chile; q Millennium Institute for Research on Depression and Personality (MIDAP), Santiago, Chile; r Department of Psychology, Universidad Loyola Andalucía, Spain; s Departamento de Psiquiatria, Faculdade de Medicina FMUSP, Universidade de São Paulo, São Paulo, Brazil; t Laboratório de Psicopatologia e Terapêutica Psiquiátrica LIM-23, Instituto de Psiquiatria, Hospital das Clinicas HCFMUSP, Universidade de São Paulo, São Paulo, Brazil; u Melbourne School of Psychological Sciences, University of Melbourne, Parkville, Australia

**Keywords:** Internet-based interventions, Perinatal depression, Guided interventions, Self-guided interventions, Blended interventions, Individual participant data meta-analysis

## Abstract

**Background:**

Internet-based interventions for perinatal depression reduce depressive symptoms in new and expectant mothers. However, modifiers of their effects, and prognostic factors of adherence, are unknown.

**Objective:**

To assess modifiers of effectiveness and deterioration, and prognostic factors of adherence in internet-based interventions for perinatal depression.

**Methods:**

Systematic searches were conducted in three bibliographic databases on February 7, 2025. Two independent researchers identified randomized controlled trials (RCTs) of internet-based interventions for perinatal depression. We conducted conventional and two-stage individual participant data meta-analysis (IPDMA).

**Results:**

Eighteen studies were eligible, and data from nine (*n* = 1094 participants) were pooled in IPDMA. We found evidence of a difference in post-treatment depressive symptoms between participants in the internet-based intervention and controls (*SMD* = 0.29 [95%CI: 0.03–0.54], small effect size), increase in response (*RR* = 1.97 [95%CI: 1.57–2.47]) and remission (*RR* = 1.73 [95%CI: 1.38–2.18]). The interventions were effective for anxiety symptoms (*SMD* = 0.26 [95%CI: 0.11–0.40]) but not for reliable deterioration (*OR* = 0.80 [95%CI: 0.49–1.32]). No modifiers of effectiveness or deterioration, or prognostic factors of adherence were identified. Baseline depressive symptoms and comorbid anxiety were prognostic factors for higher depressive symptoms at post-treatment.

**Conclusions:**

Internet-based interventions for perinatal depression are effective for depressive and anxiety symptoms in new and expectant mothers. They may be offered to patients regardless of their age, education, relationship status, employment, baseline depressive or anxiety symptoms, previous treatment, number of children, or ethnic minority status, if they wish to follow them. RCTs should collect more information on factors relevant to the perinatal period, such as partner's mental health and pregnancy complications.

**Study registration:**

Open Science Framework (https://osf.io/98tms).

## Highlights

• Internet-based interventions reduce perinatal depressive and anxiety symptoms.
• They may also prevent deterioration, but findings depend on its definition.
• Among tested variables, no modifiers of these effects were found.
• No tested variables were prognostic factors of adherence.
• Baseline depression and anxiety predict higher post-treatment depressive symptoms.

## Abbreviations

CBT cognitive behavioral therapy

EPDS Edinburgh Postnatal Depression Scale

iCBT Internet-based cognitive behavioral therapy

IPD Individual participant data

IPDMA Individual participant data meta-analysis

IPD-NMA Individual participant data network meta-analysis

ITT intention to treat

MA meta-analysis

MICE multivariate imputation by chained equations

OR odds ratio

PRISMA-IPD Preferred Reporting Items for Systematic Review and Meta-Analyses of Individual Participant Data Statement

RCI reliable change index

RCT randomized controlled trial

RR relative risk

SMD standardized mean difference

## 1 Introduction

Internet-based psychological interventions have been found to effectively reduce depressive symptoms in mothers in the perinatal period, defined as pregnancy to one year postpartum (Ashford et al., 2016; Hanach et al., 2021; Li et al., 2023; Tsai et al., 2022). These interventions cover both web-browser- and mobile-based interventions using access to the internet (Kaiser et al., 2021). Users may be guided through the therapeutic content by a therapist or a trained e-coach, through writing, audio- or video-calling. In self-guided interventions, on the other hand, no person-to-person therapeutic guidance is provided, and the user thus works through the content on their own. Technical support, automated feedback or human-supported encouragement solely focused on continuation through the modules may, however, be present (Karyotaki et al., 2021; Tong et al., 2024). Finally, some internet-based interventions combine digital modules with face-to-face (both in-person or online) sessions within the same treatment protocol. Such formats are then called blended treatments (Kooistra et al., 2020). All these formats may contribute to the accessibility of psychotherapy thanks to the reduced stigma associated with their anonymity, as well as reduced time demands on both therapist and client (Massoudi et al., 2019; Paganini et al., 2018).

Despite the considerable advantages of internet-based interventions, several questions crucial to scaling up of mental health care for new and expectant mothers with perinatal depression, using these interventions, remain unanswered. Effect sizes reported in randomized controlled trials (RCTs), ranging from no (Sawyer et al., 2019), through small and moderate (Danaher et al., 2023), to large effects (Milgrom et al., 2021; O'Mahen et al., 2014), have previously been pooled in several meta-analyses (Lau et al., 2021; Lau et al., 2022; Loughnan et al., 2019). However, these syntheses reflected certain methodological decisions hampering the possibility to fully estimate the effectiveness of internet-based interventions for perinatal depression: For example, not distinguishing between the effects of preventing onset of perinatal depression and effects of treating already existing perinatal depressive symptoms (Chae and Kim, 2021; Lau et al., 2022; Nair et al., 2018). Furthermore, some meta-analyses decided to focus on mental health of new and expectant mothers in general, without distinguishing among symptoms such as depressive, anxiety, and post-traumatic stress symptoms, distress or insomnia in the eligibility criteria of included participants, in outcome measures, or both (Lau et al., 2021; Lewkowitz et al., 2024; Li et al., 2023; Loughnan et al., 2019). This makes it difficult to disentangle these effects and complicates conclusions for society-wide policy measures. Finally, the most comprehensive meta-analysis of trials on psychological interventions of perinatal depression to date (Cuijpers et al., 2023a), including 49 comparisons of individual, group, and internet-based psychological interventions, did not investigate the effects of self-guided internet-based treatments.

It also remains unclear whether participant-level factors, for example socio-demographic or clinical characteristics, influence the responsiveness to internet-based interventions for perinatal depression. Individual participant data meta-analysis (IPDMA) is an advantageous methodology to identify prognostic factors (i.e., characteristics implying that an individual is more likely to improve, regardless of treatment) (Riley et al., 2019) or effect modifiers (i.e., characteristics predicting that an individual is more likely to benefit from one treatment, rather than another) (Noma et al., 2019). Such methodology tackles the problem of RCTs which often lack the statistical power to assess moderators, by combining primary data of multiple RCTs and analyzing them together (Cuijpers et al., 2022). Yet, no IPDMA has so far been conducted to identify prognostic factors and modifiers of effectiveness of internet-based interventions for perinatal depression. In several IPDMAs, participants with higher baseline depression severity benefitted more from internet-based interventions (Bower et al., 2013; Karyotaki et al., 2018a; Kuyken et al., 2016; Reins et al., 2021). In a network IPDMA (IPD-NMA) focusing on the effectiveness of internet-based interventions for depression in general adult samples, participants with mild severity benefitted equally from self-guided and guided internet-based cognitive behavioral therapy (iCBT), but participants with higher baseline depression severity benefitted from guided iCBT more. Furthermore, more severe depression at baseline and not being employed were shown to be prognostic factors, associated with higher symptom severity at post-treatment (Karyotaki et al., 2021). However, these IPDMAs did not focus on perinatal depression, and some characteristics relevant to this target population, such as number of children (Gilson et al., 2018), could not be assessed as potential effect modifiers.

Some participants undergoing psychological interventions may deteriorate, meaning that they experience increase instead of decrease of symptoms (Cuijpers et al., 2021). However, negative effects are often not sufficiently reported for internet-based interventions (Etzelmueller et al., 2020). This may, in turn, result in their insufficient implementation due to concerns about their advantages and disadvantages (Andersson and Titov, 2014; Karyotaki et al., 2015). While no prognostic factors or effect modifiers were observed for deterioration in internet-based interventions in general (Karyotaki et al., 2018b), it is not clear whether certain subgroups of individuals with perinatal depression exist who are particularly vulnerable for deterioration when undergoing internet-based interventions.

Finally, another under-researched topic is adherence to internet-based interventions for perinatal depression. While it has been described that effects of internet-based interventions in general are impeded by low treatment adherence (Donkin et al., 2011; Karyotaki et al., 2017), adherence to internet-based interventions for perinatal depression is sometimes as low as 13% (Davis et al., 2022). In previous IPDMAs focused on general adult population, lower adherence to internet-based interventions was found in younger, male, lower-educated (Karyotaki et al., 2015; Tong et al., 2026), employed participants (Tong et al., 2026), and participants with comorbid anxiety (Karyotaki et al., 2015). Evidence is lacking for the very specific target group with perinatal depression, even though understanding prognostic factors of adherence would enable us identify individuals vulnerable to drop out and provide them with support when needed.

While we may expect some effect modifiers in interventions for general and perinatal depression to be similar, it is crucial to explore them in perinatal depression in particular (Batt et al., 2020). Perinatal depression is associated with hormonal changes (Frieder et al., 2019) and unique psychosocial stressors, such as worries about pregnancy, (manner of) delivery, or care for the baby (Venkatesh et al., 2014). Moreover, treatments for perinatal depression occur in the wider context of health care related to childbirth and infant care, often focusing not only on depressive symptoms, but also on improving maternal self-efficacy (Batt et al., 2020) and addressing new situations, such as body changes (Branquinho et al., 2024) and sleep deprivation (Milgrom et al., 2021). Information about effect modifiers of internet-based interventions for perinatal depression, and prognostic factors of adherence to them, could enable us to offer to new and expectant mothers the most suitable intervention based on their characteristics, and improve implementation of such interventions (Karyotaki et al., 2022). This would in turn contribute to reducing the deleterious secondary consequences perinatal depression has on the whole family unit, including well-being of the mother, partner's mental health, and development of the child (Atkinson et al., 2021; Fekadu Dadi et al., 2020). Therefore, the current study aimed to identify socio-demographic and clinical modifiers and prognostic factors of (1) effectiveness, (2) deterioration, and (3) adherence, in internet-based treatments for perinatal depression.

## 2 Methods

The Preferred Reporting Items for Systematic Review and Meta-Analyses of Individual Participant Data (PRISMA-IPD) Statement (Stewart et al., 2015) was applied when conducting and reporting the analyses of this study (see Appendix A for the Checklist). The protocol of the current project was registered in the Open Science Framework (https://osf.io/98tms). The current sub-study focuses on effect modifiers in treatment of perinatal depression, the results of the registered sub-study on effect modifiers in prevention of perinatal depression will be reported elsewhere. For minor deviations from the registered protocol and their reasons, please, see Appendix B.

### 2.1 Selection and inclusion of studies

An existing individual participant data (IPD) meta-analytic database focused on internet-based interventions for the treatment of depression and/or anxiety (https://osf.io/p5emr/) was used to identify eligible trials. This database was created by systematic searches in three bibliographic databases (PubMed, PsycINFO, EMBASE) on the 7th of February 2025, and no language restrictions were applied. Appendix C shows used search strings. All titles, abstracts, and full texts were screened by two independent researchers (either MC, OP, or LT), who resolved disagreements through discussion or consultation with a senior researcher (EK). Potentially eligible trials were also requested from other authors in the field, found through reference checks in published meta-analyses, and through searching in Metapsy, a living meta-analytic database on psychological interventions for mental health (https://osf.io/825c6/) (Cuijpers et al., 2019).

In the current study, RCTs were included if they: (1) compared internet-based (i.e., blended, guided, or self-guided) psychological intervention; (2) to a control condition (such as care-as-usual, waitlist control, or attention control) or to another internet-based intervention (blended, guided, or self-guided), (3) targeted depressive symptoms; and (4) included new or expectant mothers (from pregnancy to one year postpartum) with depression (both based on a diagnostic interview or a cut-off of at least mild depression on a self-report measure). We did not apply any restrictions in terms of mental or physical comorbidities. However, we excluded studies that fulfilled criteria of telehealth, rather than internet-based interventions (such as interventions only via videoconferencing or telephone in real time), or computerized interventions not requiring internet (such as self-help books delivered on a CD-ROM). Finally, we did not include universal or selective prevention trials, meaning studies in which not all participants reported at least mild depressive symptoms based on a diagnostic interview or a cut-off on a self-report measure, as in the current study, we are only focusing on treatment.

### 2.2 Collection and harmonization of data

Study-level characteristics were collected from the publications (Appendix D) by two independent reviewers (either MC, OP, LT, CM, or IF), and participant-level characteristics from the authors of the studies (Appendix E). Corresponding authors of each eligible trial were contacted first, and if they did not respond within 2 weeks, we sent a reminder. Then, additional authors of the studies were contacted via e-mail or platforms such as LinkedIn or ResearchGate. A study was considered unavailable and thus excluded from the IPDMA if none of the authors responded to 3 attempts. All received IPD were harmonized, so that the variable names and categories matched, following a standardized publicly accessible coding manual (Karyotaki et al., 2023).

### 2.3 Assessment of risk of bias

Risk of Bias was assessed by two independent reviewers (either MC, OP, LT, CM, or IF) using the Cochrane Risk of Bias tool 2.0 (Higgins et al., 2019), who consulted any disagreements with each other or with a senior researcher (EK). The tool involves evaluation of risk of bias in five domains, namely due to (1) the randomization process, (2) deviations from intended interventions, (3) missing outcome data, (4) measurement of the outcome, and (5) selection of the reported results. Each domain was assessed as low or high risk of bias, or some concerns, and a conclusion was made for each study based on the assessment of the domains. For the conventional meta-analysis (MA), studies were evaluated based on the information reported in the published articles. For the IPDMA, assessment was conducted according to the completeness of received data. For example, a study could be evaluated as low risk in domain 5 if the authors provided data for all pre-registered outcomes, even if it was evaluated as high-risk for the conventional MA due to lack of reporting of all pre-registered outcomes. Furthermore, a study was assessed as high risk in domain 3 if dropout was 50% and more, or different by at least 30% between the two arms (Karyotaki et al., 2021). In addition, we evaluated the strength of the evidence of our main findings using the GRADE assessment (Schünemann et al., 2013).

### 2.4 Statistical analysis

The analyses were conducted using the packages “mice”, “miceadds” (Robitzsch et al., 2020), “mitml” (Grund et al., 2021), “brms” (Bürkner, 2017), “betareg” (Zeileis et al., 2016), “stdReg” (Sjolander and Dahlqwist, 2021), “purrr” (Mailund, 2022), “metapsyTools”, version 1.0.12 (Harrer et al., 2022), “meta” (Schwarzer, 2007), and “lme4” (Bates et al., 2015) in R (version 4.4.3) and RStudio (version 2026.01.0 + 392). We applied the conventional significance threshold of *p* < .05 throughout the analyses.

We first compared whether effects reported in studies which did and did not provide IPD differed via a subgroup analysis of a conventional MA. In a random-effects model, we calculated Hedges' *g* (Hedges and Olkin, 2014), and we further investigated heterogeneity by calculating the *I*^*2*^-statistic with 95% CIs (Higgins et al., 2003). We assessed publication and small study bias using Egger's test of intercept (Egger et al., 1997), and adjusted for it using the trim-and-fill procedure (Duval and Tweedie, 2000), limit meta-analysis method (Rücker et al., 2011), and the selection model (Carter et al., 2019; McShane et al., 2016). Appendix F shows more details about the main and sensitivity analyses.

Analyses of effectiveness and deterioration were conducted according to the intention-to-treat (ITT) principle. Per-study and per-arm (Harrer et al., 2023) multivariate imputation by chained equations (MICE) (van Buuren et al., 2015) based on the missing-at-random assumption was conducted to handle missing data in the outcomes and variables tested as effect modifiers. Analyses conducted in the *m* = 50 multiply imputed datasets were then combined using Rubin's rules (Rubin, 2018). The imputation matrices and more details about MICE can be seen in Appendix G. On the contrary, adherence data were based on usage logs instead of self-reported, and therefore objective. Thus, for the adherence analysis, we used only data of participants for whom information about adherence was available and did not impute missing data, as no strong assumptions were present for the reason why this data was missing. This is in line with previous investigations of prognostic factors of adherence (Karyotaki et al., 2015; Tong et al., 2026).

In the IPDMA, effectiveness was measured as reduction in depressive symptom severity expressed on the scales used most often by the included studies, converted to common metrics (Choi et al., 2014; Wahl et al., 2014) by R package *ipdconverters* (Harrer et al., 2025). To test effects of the interventions on reduction in anxiety symptoms as secondary outcome, anxiety scales were standardized using z-scores, as no common metrics were available for most outcomes. *Z*-scores were created within each study by subtracting the mean from the score of the individual participant, and dividing this number by the standard deviation (Andrade, 2021), an approach in line with previous IPDMAs (Büscher et al., 2020). Deterioration was delineated as clinically significant deterioration according to the reliable change index (RCI) (Jacobson and Truax, 1992), a method ensuring that the deterioration cannot be attributable to a measurement error (Barkham et al., 2021), where participants showing an increase in their score of RCI of +1.96 are classified as having deteriorated clinically significantly (Jacobson and Truax, 1992). Adherence was defined as an outcome reflecting engagement with the treatment, namely the percentage of modules completed at post-treatment (meaning number of modules completed divided by the number of modules offered) and calculated for intervention arms only (i.e., not as a mediator of treatment effectiveness).

The IPDMA was conducted using a two-stage approach. This was done because two-stage approaches minimize the risk of aggregation bias due to model misspecification (Riley et al., 2021), while yielding equivalent results to (correctly specified) one-stage models (Morris et al., 2018). In the first stage, effectiveness was explored using two-way ANCOVAs, where we calculated the effect of an internet-based intervention in comparison to a control condition (independent variable) on post-treatment depressive symptoms (dependent variable), while controlling for baseline depressive symptom severity (covariate), which helped us reduce the unexplained variation within the groups (Harrer et al., 2023). The linear model underlying ANCOVA provided estimated *β*-coefficients of their treatment effect and its 95% CIs, informing us how many units the dependent variable changes per each unit of change in the independent variable (the higher the *β*, the larger the effect). The *β*-coefficients were then pooled in a traditional frequentist random-effects meta-analysis (Harrer et al., 2021), and standardized mean difference (SMD) was calculated from the resulting pooled *β*. For depressive symptoms, we divided the *β* estimates (i.e., the difference in scores at post-treatment between the two treatments) by the population standard deviation of the sample at endpoint (*SD* = 10 by definition in common metrics (Wahl et al., 2014)). For anxiety symptoms, we considered *β* estimates equal to SMD, because they were based on z-standardized dependent and independent variable (Nieminen, 2022). Small, moderate and large effect sizes are expressed as *SMD* = 0.20, 0.50, and 0.80, respectively (Cohen, 2013).

Deterioration was analyzed using Bayesian logistic regression (Gelman et al., 1995), because it was a rare event in all studies (Klüppelberg et al., 2014), which lead to persistent convergence issues with frequentist methods. We used weakly informative normal priors (Harrer et al., 2021; Williams et al., 2018) to support convergence of the Bayesian model (Lemoine, 2019). Bayesian meta-analysis with a half-Cauchy prior (0, 0.5) for between study heterogeneity (*τ*^*2*^) (Harrer et al., 2021) then provided odds ratios (*OR*s) for this outcome (Cuijpers, 2016), which show the odds of an outcome being higher (lower) in the intervention as opposed to control group.

Logit-link beta regression models were used to analyze adherence data, as adherence was expressed on a scale between zero and one and showed strong deviations from normality (Ferrari and Cribari-Neto, 2004). Extreme values were shifted into the open (0,1) range. This analysis again provided *β*s and their 95% CIs, which were then pooled in a traditional frequentist meta-analysis (Harrer et al., 2021). See Appendix H for additional details about the IPDMA analyses.

Baseline participant-level characteristics were then assessed as potential modifiers of effects and deterioration in internet-based interventions for perinatal depression, and prognostic factors of adherence to them: baseline depression severity (continuous), age (continuous), education (university vs lower), relationship status (yes vs no), employment status (part- or full-time job or not), baseline anxiety symptoms (continuous), comorbid anxiety (yes or no), previous psychotherapy (yes or no), number of children (continuous), and ethnic minority status (yes or no). All variables were tested individually, while controlling for baseline depressive symptoms. No corrections were applied, as no hypothesis was tested and the analysis was only of an exploratory nature (Armstrong, 2014). We pooled data if available in at least 5 studies, for 500 participants for effect modifiers and 100 for prognostic factors, because sufficient data is required to avoid parameter estimation uncertainty (Chandler et al., 2019; Riley et al., 2020; Valentine et al., 2010), and 5 datasets are commonly used to assess effect modifiers in current IPDMAs (Godolphin et al., 2024).

## 3 Results

### 3.1 Selection and inclusion of studies

Appendix I shows the flow chart of the systematic search and study inclusion. We found 25,989 studies in the searches, and while 1798 full texts were screened, 18 studies were eligible. Ten studies (55.6%) with 1128 participants (585 in the intervention and 543 in the control conditions) provided their IPD. For the conventional MA, 18 studies (10 which did and 8 which did not contribute with their data, mainly due to no response to our attempts of contact) provided aggregated data in their published reports. Only one study (Branquinho et al., 2024) compared guided intervention to blended, and it was therefore not possible to pool its data with other studies in either conventional or IPDMA due to the lack of a common comparator (although the authors provided IPD). This study is still included in Table 1, Table 2, which show characteristics of the studies and interventions, respectively. References of pooled studies are presented in Appendices J (conventional MA) and K (IPDMA).

**Table 1:** Study Characteristics.

Study	Country	Age (*M*)	Gestational age (weeks at baseline)	Diagnosis	Conditions (*n*)a)	Study Results
Branquinho, 2024⁎	Portugal	32.1	Postpartum *M* = 5.45 months	At least 4 symptoms of MDE, at least one being dysphoria or anhedonia + cut-off (EPDS >9)	1. Blended CBT (17) 2. Guided CBT (17)	The two formats did not differ in reducing depressive (*F* = 4.220, *p* = .05), anxiety symptoms (*F* = 0.120, *p* = .73), or in reliable improvement (*χ*^2^ = 0.057, *p* = 1.00) at post-treatment.
Dai, 2025	China	30.0	Pregnant *M* = 34.07	Cut-off (EPDS ≥10)	1. CBT (45) 2. CAU (41)	The two arms did not score significantly differently at post-treatment in EPDS (*p* = .253) or PHQ-9 scores (*p* = .116), but they differed in breastfeeding time in months at the follow-up (6 months after delivery; *p* = .001). The intervention group breastfed longer (*M* = 5.52; *SD* = 1.25). than the control group (*M* = 4.50; *SD* = 1.69).
Danaher, 2022⁎	USA	31.9	50% pregnant (26–28 weeks) 50% postpartum (<12 months)	Cut-off (EPDS >12)	1. CBT (96) 2. CAU (95)	The intervention showed greater decreases than the control group in depression severity (*b* = −0.76, *SE* = 0.25, *t* = −3.03, *p* = .003, *d* = −0.40) and stress (*b* = −0.51, *SE* = 0.22, *t* = −2.37, *p* = .019, *d* = −0.36), but not in anxiety (*b* = −0.18, *SE* = 0.17, *t* = −1.08, *p* = .283, *d* = −0.13).
Fonseca, 2020⁎	Portugal	32.6	Postpartum *M* = 2 months	Cut-off (EPDS >10)	1. CBT (98) 2. WL (96)	Mothers in the intervention group showed a larger decrease than the control group in depressive (*b* = −1.18, *SE* = 0.58, *p* = .043) and anxiety symptoms (*b* = −1.23, *SE* = 0.52, *p* = .019) at post-treatment, but not in dyadic satisfaction (*b* = −0.06, *SE* = 0.09, *p* = .509).
Forsell, 2017⁎	Sweden	31.0	Pregnant *M* = 17.19	MDD (SCID-I) + MADRS-S = 15–35	1. CBT (22) 2. WL (20)	The intervention group reported lower MADRS-S depressive symptoms at post-treatment (*F* = 15.31, *p* < .001, *g* = 1.21) and were more likely to achieve a statistically reliable improvement (*RR* = 0.36, *p* = .004) than the control group. However, the difference in the EPDS scores (*F* = 1.06, *p* = .31, *g* = 0.52), anxiety (*F* = 2.81, *p* = .10, *g* = 0.63), and insomnia severity (*F* = 1.94, *p* = .17, *g* = 0.44) was not significant.
Franco, 2024⁎	Chile		Postpartum *M* = 3.54 months	MDD or minor depressive disorder (MINI) + (PHQ-9 ≥ 5)	1. CBT (33) 2. WL (32)	At post-treatment, there were no differences between the arms in the presence a diagnose of depression, or in depressive symptoms as measured by both PHQ-9 or EPDS (*F* = −1.70, *p* > .05; *F* = 2.63, *p* > .05).
Hassdenteufel, 2023	Germany	32.6	Pregnant *M* = 21.2	Cut-off (EPDS >9)	1. 3rd (142) 2. CAU (174)	Anxiety symptoms at post-treatment were not significantly lower in the intervention than the control group (*p* > .122), however, the rate of women at risk for postpartum depression (*F* (1,149) = 5.334, *p* = .022) was lower in the intervention than the control group.
Jannati, 2020	Iran	27.5	Postpartum (<6 months)	Cut-off (EPDS ≥13)	1. CBT (39) 2. WL (39)	Depressive symptoms were lower in the intervention than in the control group at post-treatment (*p* < .001).
Milgrom, 2016⁎	Australia	31.6	Postpartum *M* = 6.33 months	MDD or minor depressive disorder (SCID-IV) + EPDS = 11–23	1. CBT (21) 2. CAU (22)	At post-treatment, the intervention group no longer met diagnostic criteria for depression, more often than the control group (79% vs 18%, *χ*^2^ = 10.3, *p* = .001), and they also showed a larger improvement in depressive (*F* (1,40) = 7.4, *p* = .01, *d* = 0.83) and anxiety symptoms (*F* (1,40) = 153, *p* = .01, *d* = 0.83).
Milgrom, 2021⁎	Australia	32.1	Postpartum *M* = 6.76 months	MDD or minor depressive disorder (SCID-IV) + EPDS = 11–25	1. CBT (39) 2. CAU (38)	The intervention group remitted from the depressive episode more often than the control group (78% vs 58%). The intervention group was also more effective in reducing depressive (*t* (124) = −2.61, *p* = .01) and anxiety (*t* (124) = −2.01, *p* = .05) symptoms, and stress (*t* (124) = −2.78, *p* = .006).
O'Mahen, 2013a	United Kingdom	NR	Postpartum (<12 months)	MDD (ICD-10) + EPDS >12	1. BAT (37) 2. CAU (34)	The intervention group reported lower depressive (*d* = −0.96, *p* < .01) and anxiety symptoms (*d* = −0.59, *p* < .03) at post-treatment.
O'Mahen, 2013b	United Kingdom	32.3	Postpartum (<12 months)	Cut-off (EPDS >12)	1. BAT (181) 2. CAU (162)	More mothers in the intervention than the control group scored as non-depressed at post-treatment (*OR* = 1.78, 95% CI = 1.28–2.49, *p* = .001).
Pugh, 2016⁎	Canada	30.8	Postpartum (<12 months)	Cut-off (EPDS ≥10)	1. CBT (25) 2. WL (25)	Depressive (F(1,11.82) = 5.15, *p* = .02) and anxiety symptoms (F(3,33) = 9.23, *p* < .001), stress (F(3,38) = 4.23, *p* = .01) and parental distress (F(3,35) = 19.86, *p* < .001) were reduced in the intervention group more than in the control group.
Sawyer, 2019	Australia	31.7	Postpartum (2–6 months)	Cut-off (EPDS ≥7)	1. CBT (54) 2. WL (57)	There was no significant difference in depression scores post-treatment between the intervention and the control group (*MD* = 0.7, 95%CI: −1.1–2.5).
Seo, 2022	South Korea	NR	Postpartum (<12 months)	Cut-off (EPDS ≥9)	1. CBT (37) 2. CAU (36)	There were no significant differences between the intervention and the control group in depressive symptoms (*MD* = −3.24, *SD* = 3.99, *p* = .129), social support (*MD* = 2.59, *SD* = 11.46, *p* = .448) or sleep quality (*MD* = −0.97, *SD* = 2.86, *p* = .373) at post-treatment.
Sun, 2021⁎	China	29.9	Pregnant *M* = 16.8	Cut-off (EPDS >9 or PHQ-9 > 4)	1. 3rd (84) 2. Oth control (84)	At post-treatment, depressive (*d* = 0.47, *p* = .03) and anxiety symptoms (*d* = 0.26, *p* = .01) were lower in the intervention than in the control condition, but not stress (*d* = 0.27, *p* = .94).
Vigod, 2021	Canada	33	Postpartum (<12 months)	Cut-off (EPDS ≥10)	1. IPT (37) 2. WL (40)	There was no significant difference at depression scores post-treatment between the intervention and the control group (*MD* = −0.58, 95%CI: −2.68–1.52), nor was there a difference in the number of mothers who remitted (37.8% vs 25%, *χ*^2^ = 1.48, *p* = .224).
Zuccolo, 2024⁎	Brazil	31.2	Postpartum *M* = 4.60 months	Cut-off (EPDS > 10)	1. CBT (132) 2. Psychoeducation (132)	There was no significant difference between the arms at post-treatment in depressive (*p* = .604) or anxiety symptoms (*p* = .431).

*Note.*

Abbreviations (alphabetical): 3rd: third-wave cognitive behavior therapy; 95% CI: 95% confidence interval; b = beta coefficient in regression; BAT: behavioral activation; CAU: Care as usual; CBT: cognitive behavior therapy; d = Cohen's d; EPDS: Edinburgh Postnatal Depression Scale; F: F-statistic; g = Hedges' g; ICD-10: International Classification of Diseases, 10th edition; IPT: Interpersonal Psychotherapy; M: mean; MADRS-S: Montgomery-Åsberg Depression Rating Scale—Self-rated; MD: mean difference; MDD: major depressive disorder, MDE: major depressive episode; n: number; NR: not reported; p: *p*-value; PHQ-9: The Patient Health Questionnaire-9 items; OR: odds ratio; Oth control: Other control (such as attention control); RoB: Risk of Bias; RR: relative risk; SCID-I: Structure Clinical Interview for DSM-IV, Axis I; SD: standard deviation; SE: standard error; t: t-statistic; WL: waitlist condition; *χ*^2^: chi-square-statistic.

References:

First, M. B., & Gibbon, M. (2004). The structured clinical interview for DSM-IV axis I disorders (SCID-I) and the structured clinical interview for DSM-IV axis II disorders (SCID-II). In M. Hilsenroth, & D. L. Segalaniel (Eds.), *Comprehensive handbook of psychological assessment: Vol. 2. Personality assessment* (pp. 134–143). Hoboken, NJ: Wiley.

Kroenke, K., Spitzer, R. L., & Williams, J. B. (2001). The PHQ‐9: validity of a brief depression severity measure. *Journal of general internal medicine*, *16*(9), 606-613.

Lewinsohn, P. M., Seeley, J. R., Roberts, R. E., & Allen, N. B. (1997). Center for Epidemiologic Studies Depression Scale (CES-D) as a screening instrument for depression among community-residing older adults. *Psychology and aging*, *12*(2), 277.

McCabe-Beane, J. E., Segre, L. S., Perkhounkova, Y., Stuart, S., & O’Hara, M. W. (2016). The identification of severity ranges for the Edinburgh Postnatal Depression Scale. *Journal of Reproductive and Infant Psychology*, *34*(3), 293-303.

Montgomery, S. A., & Åsberg, M. A. R. I. E. (1979). A new depression scale designed to be sensitive to change. *The British journal of psychiatry*, *134*(4), 382-389.

World Health Organization. (1993). *The ICD-10 classification of mental and behavioural disorders: diagnostic criteria for research* (Vol. 2). World Health Organization.

Sheehan, D. V., Lecrubier, Y., Sheehan, K. H., Amorim, P., Janavs, J., Weiller, E., ... & Dunbar, G. C. (1998). The Mini-International Neuropsychiatric Interview (MINI): the development and validation of a structured diagnostic psychiatric interview for DSM-IV and ICD-10. *J clin psychiatry*, *59*(Suppl 20), 22-33.

⁎ Included in the individual participant data meta-analysis.

a Number of participants refers to the number provided by the authors, if they provided IPD, the number of participants available for the conventional MA if no IPD were provided, or randomized number if it was not clear what the number of participants available for the conventional MA was.

**Table 2:** Intervention and Control Characteristics.

Study	Intervention Timing a)	Intervention Format	No. Sessions (mean/planned)	Intervention Content	Control Content
Branquinho, 2024⁎	Postnatal	Blended versus Guided (Regularly by a psychologist over the phone)	13/13	Be a Mom Coping with Depression. Blended psychotherapy: Web-based program: Maternity changes, PPD and emotions, thoughts, values, social support and interpersonal skills, the couple's relationship, relapse prevention. Sessions with the therapist (biweekly 60 min): Expectations toward motherhood, emotions, the CBT model, cognitive flexibility, recognizing negative thoughts, acceptance and defusion, thoughts questioning and self-compassion, identification of values, committed value-based actions and strategies to increase them, social support, communication skills, promotion of affection and intimity, problem-solving, relapse prevention plan.	Be a Mom Coping with Depression. Guided self-help: Web-based program: Maternity changes, PPD and emotions, thoughts, values, social support and interpersonal skills, the couple's relationship, relapse prevention. Phone feedback by the therapist (biweekly 20–30 min): Expectations toward motherhood, emotions, the CBT model, cognitive flexibility, recognizing negative thoughts, acceptance, thoughts questioning and self-compassion, identification of values, value-based actions behaviors, social network, communication skills, problem-solving, relapse prevention plan.
Dai, 2025	Ante- and postnatal	Guided (Two offline and three online sessions with a master-level nursing student)	5	Thinking Health Programme. Recognizing unhealthy thinking patters, replacing dysfunctional thoughts, behavioral activation, active listening, collaboration with the family, guided discovery, homework, mother health, mother-baby relationship, relationship with people around. Text, images, post-learning quiz questions, self-monitoring.	Enhanced usual care (standard care by midwives, who were informed about the participants' depressive symptoms and how to refer severe cases, information about gestation, maternal and mental health, child development, and where to seek help).
Danaher, 2022⁎	Ante- and postnatal	Self-guided (Technical support)	4/6	MomMoodBooster2. Behavioral activation, interruption of negative and increasing of positive thoughts, seeking support, tracking mood. Video and audio recordings, animations, editable lists.	Usual care (perinatal outpatient depression screening, referral network of community mental health providers, crisis hotline, relevant curriculum for obstetricians and nurse midwives).
Fonseca, 2020⁎	Postnatal	Self-guided (Technical support)	5 (planned)	BeaMom. Psychoeducation, managing expectations, identifying emotions, managing negative thoughts, focus on values and social support, assertive communication, managing changes in the couple relationship, identifying postpartum depression signs and asking for help.	Waitlist.
Forsell, 2017⁎	Antenatal	Guided (Regular asynchronous written feedback by a CBT-trained therapist)	5/10	Internet Delivered Cognitive Behavior Therapy. Psychoeducation, focus on social support, behavioral activation, negative automatic thoughts, cognitive biases, acceptance, problem solving, alternative thoughts, communication, management of labor anxiety, sleep hygiene, in the form of texts, assessments, homework, and worksheets.	Waitlist.
Franco, 2024⁎	Postnatal	Guided (Regular asynchronous written feedback by a clinical psychologist)	4/6	Mamá, te entiendo. Introduction to the intervention, psychoeducation on depression, how the cognitive-behavioral approach works, identifying thinking errors, cognitive restructuring techniques, problem-solving, behavioral activation, relapse prevention. Optional modules: psychoeducation on anxiety, exposure strategies, communicational skills.	Waitlist.
Hassdenteufel, 2023	Antenatal	Self-guided (Purely self-guided)	8 (planned)	Electronic Mindfulness-based Intervention. Psychoeducation and education (pregnancy, newborn care), mindfulness exercises, challenging negative thoughts, well-being resources, in the form of texts, audio and video files, personal skill box, interactive worksheets.	Usual care.
Jannati, 2020	Postnatal	Self-guided (Purely self-guided)	8 (planned)	Happy Mom. Psychoeducation, goal setting, noticing negative thoughts and challenging them, problem solving, improving social skills, relapse prevention.	Waitlist.
Milgrom, 2016⁎	Postnatal	Guided (Regularly by a psychologist/psychology trainee over the phone)	6/6	MumMoodBooster. Psychoeducation, behavioral activation, goal setting, focus on social support, communication skills, stress management, problem solving, sleep hygiene, newborn care, parental care, managing negative thoughts in the form of texts, audio, and video files, mood monitoring, peer web forum. Additional partner website.	Usual care (a health practitioner – general practitioner or maternal health nurse - notified about the depression status, and they were free to treat or to refer the participant to other services, as deemed appropriate, provided internet resources on mental health).
Milgrom, 2021⁎	Postnatal	Guided (Regularly by a psychologist/psychology trainee over the phone)	6/6	MumMoodBooster. Psychoeducation, behavioral activation, goal setting, focus on social support, communication skills, stress management, problem solving, sleep hygiene, newborn care, parental care, managing negative thoughts in the form of texts, audio, and video files, mood monitoring, peer web forum. Additional partner website.	Usual care (referral back to the general practitioner with a report of their diagnostic assessment, support, and referral to other services).
O'Mahen, 2013a	Postnatal	Guided (Regularly by an undergraduate mental health care worker over the phone)	5/6	Netmums. Behavioral activation, problem solving, psychoeducation, communication skills, sleep hygiene, challenging negative thoughts, exposure, connecting to peers in the area, chat room with peers and peer supporters.	Usual care (+ access to the peer chat room).
O'Mahen, 2013b	Postnatal	Self-guided (Support on demand)	11 (planned)	Netmums. Psychoeducation, behavioral activation, problem solving, communication skills, social support, challenging negative thoughts, relapse prevention, connecting to peers in the area, chat room with peers and peer supporters.	Usual care (+ access to the peer chat room).
Pugh, 2016⁎	Postnatal	Guided (Regular asynchronous e-mail feedback by a therapist)	6/7	Therapist-Assisted Internet-delivered Cognitive Behavior Therapy. Psychoeducation, challenging negative thoughts, behavioral activation, exposure, problem solving, relaxation, relapse prevention.	Waitlist (+ Pamphlet with psychoeducation on perinatal depression, websites to access support services).
Sawyer, 2019	Postnatal	Guided (Regular asynchronous moderation of an online forum by a trained nurse)	16 (planned)	eMums. Psychoeducation and education (newborn and maternal care), behavioral activation, problem solving, focus on attachment and social support, challenging negative thoughts, communication skills, peer forum with moderation, resources.	Usual care (a single home visit by a Child and Family Health Service nurse around 4 weeks of the infant's age, provision of information about services and general advice).
Seo, 2022	Postnatal	Self-guided (Human encouragement)	8 (planned)	Happy Mother. Psychoeducation, managing mood and negative thoughts, behavioral activation, focus on help-seeking behavior and social support, sleep hygiene.	Usual care (+ a leaflet explaining symptoms, causes, and coping methods with postpartum depression).
Sun, 2021⁎	Antenatal	Self-guided (Purely self-guided)	8 (planned)	Mindfulness training, including body scan, mindful breathing, mindful stretching, mindful meditation, and homework in the form of informal training during the day.	Medical check-up attention control (weekly contact with a clinically trained nursing assistant with experience in prenatal care via WeChat to discuss recent medical examinations, outpatient appointments, and potential inpatient care).
Vigod, 2021	Postnatal	Guided (Weekly asynchronous moderation of an online forum + live chat by a therapist)	10 (planned)	Mother Matters. Psychoeducation, focus on social support, interpersonal relationships (including with the baby, peer discussion forum with discussions about the module of the week.	Waitlist.
Zuccolo, 2025⁎	Postnatal	Self-guided (Technical support)	6 (planned)	Motherly. Behavioral activation and problem solving, emotional regulation, stress management, cognitive restructuring, sleep hygiene and relaxation, mindfulness, scheduling health-related events, registration of relevant information, access to educational content on health and child development. Text and graphics, input fields.	Psychoeducation app. Around 30 brief psychoeducational videos on mental health (depression, anxiety, stress, sleep, problem solving), mental symptom monitoring and recommendations based on responses.

*Note.*

Abbreviations (alphabetical): CBT: Cognitive behavior therapy.

⁎ Included in the individual participant data meta-analysis.

a Antenatal intervention timing: The whole intervention was delivered before the baby was born; Postnatal intervention timing: The intervention started after the birth of the baby; Ante- and postnatal intervention timing: The intervention started before the baby was born and continued, or could be continued, after the birth of the baby.

### 3.2 Study characteristics

Among the studies whose data were pooled in the IPDMA (*k* = 9, *n* = 1094, interventions: *n* = 551, controls: *n* = 543), most studies (*n* = 7, 77.8%) recruited their participants through the community, the rest used systematic screening. While five studies (55.6%) included participants exceeding a cut-off on a self-report depression measure, four (44.4%) required a diagnosis of a mood disorder. Eight studies (88.9%) investigated CBT and one third-wave cognitive behavioral therapy. Six (66.7%) interventions were delivered in their full length after the mother gave birth to the baby, two (22.2%) fully before birth, and one intervention (11.1%) could be delivered both before and after birth. Five studies focused on the comparison of guided internet-based intervention versus control; the rest (*n* = 4) applied a self-guided intervention. Two studies offered an additional website for partners of the participant. Controls included CAU (*n* = 3, 33.3%), WL (*n* = 4), attention control with medical assessments, and psychoeducation app (both *n* = 1). The studies were conducted in Australia (*n* = 2), Brazil, Canada, Chile, China, Portugal, Sweden, and USA.

### 3.3 Risk of bias

The risk of bias plots can be seen in Appendices L (IPDMA studies) and M (conventional MA). Among the studies whose data were pooled in the IPDMA, five had a low risk of bias and four had some concerns. All studies were at low risk of bias in deviations from intended intervention, missing outcome data, and measurement of the outcome, seven of bias arising from the randomization process, and six of bias in selection of the reported result. Furthermore, no issues were identified when harmonizing IPD.

### 3.4 Conventional meta-analysis (*k* = 17)

Results of conventional MA are shown in Appendix N. When pooling the data of the *k* = 17 eligible studies together (17 comparisons), internet-based interventions showed a significant difference in depressive symptoms severity in comparison with the control condition at post-treatment assessment (*g* = 0.53 [95% CI: 0.18 to 0.87], *p* = .005), with a moderate effect size and high heterogeneity (*I*^*2*^ = 84%, 95%CI: 76%–90%). Sensitivity analyses revealed very similar results, except for inclusion of only studies with low risk of bias (*k* = 4), where the effect was no longer significant (*g* = 0.41 [95% CI: −0.16 to 0.98]).

There was an indication of publication bias (Egger's test: *p* = .049). After the corrections, results of the analyses were no longer significant (e.g., *p* = .146 under the trim-and-fill method). There was no evidence of a difference between studies which did and did not provide their IPD (*p* = .683 in a subgroup analysis), also when controlled for other characteristics of the studies (*p* = .768). Furthermore, there were no differences in effects of studies testing CBT versus other types of psychotherapy, types of control, whether the intervention was guided or self-guided, whether the participants were recruited based on a diagnosis of a mood disorder or a cut-off on a self-report measure, and whether the study was conducted in a western or non-western country (all *p* > .05).

### 3.5 Participant characteristics

Among the participants for whom these data were available (IPDMA, see Appendix O for details, all females), the average age was 31.5 years, 75.9% (828/1091) completed university-level education, and 91.9% (999/1087) were in a relationship. In the studies which collected this kind of information, 64.8% (522/805) were (full- or part-time) employed and 25.2% (174/690) were an ethnic minority. Two hundred and nineteen participants (124 in the intervention and 95 in the control arms) did not complete post-treatment measures, indicating a 20% dropout rate (22.5% in the intervention and 17.5% in the control arms). The most administered measure of depression was the Edinburgh Postnatal Depression Scale (EPDS) (Cox and Holden, 2003), used in 6 studies. The means (*SDs*) were 12.9 (5.9) at baseline and 10.0 (5.5) at post-treatment in the ITT sample, indicating mild depression severity at both time points. On average, participants completed 59.4% (*SD* = 40%) of offered modules. In the intervention arm in studies that measured adherence and among participants for whom this data was available (*n* = 313, *k* = 7), only 105 (33.5%) participants completed all the offered modules, while 172 (55.0%) completed less than 75% of the modules.

### 3.6 Individual participant data meta-analysis (*k* = 9)

The findings of the main, two-stage IPD analysis on depressive symptom severity are shown in Table 3 and Appendix P. At post-treatment, there was evidence of a greater reduction in depressive symptom severity among participants in the internet-based intervention conditions as compared to control condition participants (*SMD* = 0.29 [95%CI: 0.03 to 0.54; 95%PI: −0.25 to 0.82], small effect size). The evidence had a moderate strength according to the GRADE assessment of main outcomes (Appendix Q). Heterogeneity was moderate (*I*^*2*^ = 59%, 95%CI: 14% - 80%). There was an indication of publication bias (Appendix R), where in two out of the three correction methods, effects became non-significant (e.g., *p* = .196 in the trim-and-fill method, two studies filled). Sensitivity analyses also suggested evidence of a difference between the two groups, such as in the one-stage IPDMA (*p* = .021). Internet-based interventions also had an effect on response (*RR* = 1.97 [95%CI: 1.57 to 2.47]) and remission (*RR* = 1.73 [95%CI: 1.38 to 2.18]). In addition, internet-based interventions for perinatal depression resulted in reductions in post-treatment anxiety in comparison to the control group, with a small effect size (*SMD* = 0.26 [95%CI: 0.11 to 0.40]).

**Table 3:** Modifiers of Effectiveness of Internet-Based Interventions for Perinatal Depression - Two-stage Individual Participant Data Meta-analysis (Intention-To-Treat Analysis).

	*k*	*N*	*SMD [95%CI]* *(95% PI)*	*β-coefficient* *[95% CI]*	*I*^*2*^*[95% CI]*	*p*
Main Effects: Group + Baseline Depression Severity						
Baseline depressive symptoms	9	1094		4.04 [2.77–5.31]		<0.001^⁎⁎⁎^
Group	9	1094	0.29 [0.03–0.54] (−0.25–0.82)	−2.91 [−5.45 to −0.37]	59.0 [14.3–80.3]	0.030⁎
Prognostic Factors and Effect Modifiers						
Baseline depressive symptoms X Group	9	1094		−0.83 [−2.66–0.99]	43.5 [0.0–73.9]	0.322
Age^a^						
Age (continuous)	9	1094		−0.51 [−1.12–0.10]	0.0 [0.0–64.8]	0.088
Age X Group	9	1094		0.57 [−0.33–1.47]	0.0 [0.0–64.8]	0.183
Education^a^						
Education (tertiary vs lower)	9	1094		−0.46 [−2.51–1.60]	0.0 [0.0–64.8]	0.622
Education X Group	9	1094		0.80 [−2.13–3.72]	0.0 [0.0–64.8]	0.548
Relationship status^a^						
Relationship status (no vs yes)	8	926		−0.69 [−2.82–1.44]	0.0 [0.0–67.6]	0.468
Relationship status X Group	6	807		0.57 [−5.68–6.81]	19.1 [0.0–63.9]	0.825
Employment status^a^						
Employment status (employed vs other)	6	810		0.03 [−3.90–3.96]	32.5 [0.0–72.8]	0.986
Employment status X Group	6	810		−0.52 [−5.42–4.39]	16.6 [0.0–78.8]	0.797
Baseline anxiety symptoms^a^						
Baseline anxiety symptoms (continuous)	8	1029		1.66 [−0.22–3.54]	55.2 [1.0–79.7]	0.075
Baseline anxiety symptoms X Group	8	1029		−1.49 [−3.52–0.53]	47.9 [0.0–76.8]	0.125
Comorbid anxiety^a^						
Comorbid anxiety (no vs yes)	9	1094		3.16 [0.97–5.34]	0.0 [0.0–64.8]	0.010⁎
Comorbid anxiety X Group	9	1094		−3.39 [−7.84–1.06]	45.3 [0.0–74.7]	0.117
Previous treatment^a^						
Previous treatment (no vs yes)	6	670		−0.43 [−3.86–3.01]	48.8 [0.0–79.7]	0.763
Previous treatment X Group	6	670		−0.92 [−3.21–1.38]	0.0 [0.0–74.6]	0.352
Number of children^a^						
Number of children (continuous)	5	570		−0.52 [−2.84–1.80]	37.5 [0.0–76.7]	0.567
Number of children X Group	5	570		1.14 [−1.08–3.35]	0.29 [0.0–79.3]	0.227
Minority status^a^						
Minority status (no vs yes)	6	690		0.32 [−2.29–2.93]	0.0 [0.0–74.6]	0.764
Minority status X Group	4	575		−0.27 [−5.41–4.86]	0.0 [0.0–84.7]	0.876

*Notes.* Abbreviations: 95%CI: 95% confidence Interval; k: number of studies; N: Number of observations; p: *p*-value.

X – interaction.

⁎ *p*-value < .05; ^⁎⁎^*p*-value < .01; ^⁎⁎⁎^*p*-value < .001.

a – Results after controlling for baseline depression severity and group.

### 3.7 Modifiers of effectiveness

None of the tested effect modifiers was significant (Table 3), meaning that the outcomes of the interventions were not more or less pronounced in any investigated specific participant subgroup. Furthermore, none of the study-level variables (Appendix R) were associated with the effects of the interventions. Participants with higher baseline depressive symptoms (β = 4.04 [95%CI: 2.77 to 5.31]) and comorbid anxiety (β = 3.16 [95%CI: 0.97 to 5.34]) tended to report higher depressive symptoms at post-treatment, regardless of the condition that they were assigned to.

### 3.8 Modifiers of deterioration

Results of the deterioration analysis are shown in Table 4. We found no evidence that internet-based interventions for perinatal depression led to significantly lower clinically reliable deterioration (*OR* = 0.80 [95%CI: 0.49 to 1.32]), but when deterioration was defined as any increase in the score from baseline to post-treatment, participants in intervention groups were less likely to deteriorate (*RR* = 3.12 [95%CI: 2.55 to 3.81]). We did not find any modifiers of these effects, meaning that no investigated subgroup of participants was more or less likely to deteriorate if they received the intervention than other subgroups.

**Table 4:** Modifiers of Reliable Deterioration in Internet-Based Interventions for Perinatal Depression - Two-stage Individual Participant Data Meta-analysis (Intention-To-Treat Analysis).

	*k*	*N*	*OR [95% CrI]* *(95% PI)*	*Heterogeneity*
Main Effects: Group + Baseline Depression Severity				
Baseline depressive symptoms	9	1094	0.68 [0.49–0.96]⁎ (0.40–1.18)	*τ*^*2*^ = 0.26, 95%CrI: 0.01–0.78
Group	9	1094	0.80 [0.49–1.32] (0.35–1.87)	*τ*^*2*^ = 0.16, 95%CrI: 0.00–0.48
Prognostic Factors and Effect Modifiers				
Baseline depressive symptoms X Group	9	1094	0.83 [0.53–1.36] (0.36–2.08)	*τ*^*2*^ = 0.28, 95%CrI: 0.01–0.80
Age^a^	9	1094		
Age (continuous)			0.94 [0.64–1.42] (0.39–2.42)	*τ*^*2*^ = 0.31, 95%CrI:0.01–0.85
Age X Group			1.09 [0.70–1.75] (0.50–2.40)	*τ*^*2*^ = 0.24, 95%CrI: 0.01–0.71
Education^a^	9	1094		
Education (tertiary vs lower)			0.72 [0.42–1.23] (0.33–1.58)	*τ*^*2*^ = 0.21, 95%CrI:0.01–0.66
Education X Group			0.90 [0.49–1.63] (0.38–2.06)	*τ*^*2*^ = 0.23, 95%CrI: 0.01–0.73
Relationship status^a^	8	926		
Relationship status (no vs yes)			0.91 [0.50–1.67] (0.39–2.11)	*τ*^*2*^ = 0.24, 95%CrI: 0.01–0.74
Relationship status X Group			0.75 [0.42–1.35] (0.32–1.81)	*τ*^*2*^ = 0.24, 95%CrI: 0.01–0.75
Employment status^a^	6	810		
Employment status (employed vs other)			1.28 [0.68–2.44] (0.50–3.25)	*τ*^*2*^ = 0.27, 95%CrI: 0.01–0.91
Employment status X Group			0.78 [0.40–1.52] (0.30–2.09)	*τ*^*2*^ = 0.27, 95%CrI: 0.01–0.88
Baseline anxiety symptoms^a^	8	1029		
Baseline anxiety symptoms (continuous)			1.27 [0.81–1.93] (0.55–2.79)	*τ*^*2*^ = 0.26, 95%CrI: 0.01–0.78
Baseline anxiety symptoms X Group			0.79 [0.50–1.30] (0.37–1.79)	*τ*^*2*^ = 0.24, 95%CrI: 0.01–0.72
Comorbid anxiety^a^	9	1094		
Comorbid anxiety (no vs yes)			1.23 [0.72–2.12] (0.55–2.66)	*τ*^*2*^ = 0.22, 95%CrI: 0.01–0.68
Comorbid anxiety X Group			0.84 [0.49–1.45] (0.38–1.85)	*τ*^*2*^ = 0.22, 95%CrI: 0.01–0.68
Previous treatment^a^	6	670		
Previous treatment (no vs yes)			0.91 [0.44–1.88] (0.33–2.59)	*τ*^*2*^ = 0.29, 95%CrI: 0.01–0.99
Previous treatment X Group			0.84 [0.39–1.77] (0.29–2.38)	*τ*^*2*^ = 0.29, 95%CrI: 0.01–1.02
Number of children^a^	5	570		
Number of children (continuous)			0.89 [0.45–1.73] (0.28–2.84)	*τ*^*2*^ = 0.33, 95%CrI: 0.01–1.08
Number of children X Group			0.95 [0.49–1.84] (0.31–2.86)	*τ*^*2*^ = 0.33, 95%CrI: 0.01–1.00
Minority status^a^	6	690		
Minority status (no vs yes)			1.02 [0.50–2.08] (0.37–1.04)	*τ*^*2*^ = 0.27, 95%CrI: 0.01–0.90
Minority status X Group			0.88 [0.44–1.82] (0.32–2.56)	*τ*^*2*^ = 0.28, 95%CrI: 0.01–0.88
Sensitivity analysis (any deterioration)	9	1094	***RR [95% CI] (95% PI)***	
Group			3.12 [2.55–3.81]⁎ (2.46–3.95)	*I*^*2*^ = 0.0, 95%CI: 0.0–64.8

*Notes.* Abbreviations: 95%CI: 95% confidence interval; 95% CrI: 95% credible Interval; 95%PI: 95% prediction interval; k: number of studies; N: Number of observations; OR: Odds ratio.

X – interaction

⁎ - Evidence of a true association.

a – Results after controlling for baseline depression severity and group.

### 3.9 Prognostic factors of adherence

Table 5 shows the analysis of prognostic factors of adherence. Among the available variables and within the available data, no statistically significant prognostic factors were shown to be related to treatment adherence in either main or in sensitivity analyses (Appendix S).

**Table 5:** Prognostic Factors of Adherence to Internet-Based Interventions for Perinatal Depression - Two-stage Individual Participant Data Meta-analysis (Complete Case Analysis).

	*k*	*N*	*β-coefficient [95% CI] (95% PI)*	*I*^*2*^ [95% CI]	*p*
Baseline depressive symptoms (continuous)	7	313	−0.08 [−0.34–0.18] (−0.59–0.44)	44.3 [0.0–76.6]	0.503
Age (continuous)	7	310	0.08 [−0.10–0.27] (−0.10–0.27)	0.43 [0.0–70.9]	0.312
Education (tertiary vs lower)	6	297	−0.25 [−0.62–0.11] (−0.80–0.29)	0.0 [0.0–74.6]	0.134
Relationship status (no vs yes)	4	161	0.30 [−0.85–1.45] (−1.14–1.75)	0.0 [0.0–84.7]	0.463
Employment status (employed vs other)	4	232	0.05 [−0.33–0.43] (−0.67–0.76)	0.0 [0.0–84.7]	0.722
Baseline anxiety symptoms (continuous)	5	257	0.11 [−0.24–0.45] (−0.50–0.71)	33.9 [0.0–75.0]	0.441
Comorbid anxiety (no vs yes)	7	299	−0.04 [−0.54–0.46] (−0.90–0.81)	23.1 [0.0–65.9]	0.846
Previous treatment (no vs yes)	5	198	−0.02 [−0.40–0.37] (−0.54–0.51)	0.0 [0.0–79.2]	0.911
Number of children (continuous)	3	141	−0.10 [−0.24–0.04] (−0.56–0.37)	0.0 [0.0–89.6]	0.096

*Notes.* Abbreviations: 95%CI: 95% confidence Interval; 95% PI: 95% prediction Interval; k: number of studies; N: Number of observations; p: *p*-value; SE: Standard error.

Data for the studies by Danaher and Zuccolo were not available.

^⁎^*p*-value < .05.

^⁎⁎^*p*-value < .01.

^⁎⁎⁎^*p*-value < .001.

## 4 Discussion

The current IPDMA focused on modifiers of effects of internet-based interventions for perinatal depression, and prognostic factors of adherence to them. We analyzed data from 9 RCTs (18 in the qualitative overview and 17 in the conventional MA). There was evidence for effectiveness of internet-based interventions for perinatal depression in reduction of depressive symptoms (with small effect size), and for higher response and remission rates in the intervention study arms. They further seemed effective for anxiety symptoms (with small effect size), and any deterioration, but not reliable deterioration. Among the tested variables, we did not find any modifiers of effects, or prognostic factors of adherence. Only baseline depressive symptoms and comorbid anxiety were associated with higher post-treatment depressive symptoms, regardless of receiving treatment or not.

Our finding of a difference in depressive symptom severity at post-treatment between the intervention and control groups is in line with previous meta-analyses on the effectiveness of internet-based interventions for perinatal depression (Chae and Kim, 2021; Lau et al., 2021; Lau et al., 2022; Li et al., 2023; Loughnan et al., 2019; Nair et al., 2018; Tsai et al., 2022). This result holds despite methodological differences from previous studies, and despite variation in individual participant characteristics. Therefore, there seem to be no specific individuals in terms of age, education, relationship status, employment, baseline depressive or anxiety symptoms, comorbid anxiety, previous treatment, number of children, or ethnic minority status, in whom these interventions would be expected to have weaker or stronger effects in comparison to other individuals.

We observed a small effect on depressive and anxiety symptoms. These effects are slightly less encouraging than results of a previous conventional meta-analysis on all formats of psychological treatments for perinatal depression (Cuijpers et al., 2023a), which reported a moderate effect size of *g* = 0.67 [95% CI 0.45 to 0.89], thus not differing from the moderate effect size of our own conventional MA (*g* = 0.53 [95% CI: 0.18 to 0.87]). The discrepancy with our IPDMA may be caused by the fact that conventional MAs cannot control whether included data are completers-only or are based on the ITT principle. If the latter, it further cannot influence the way missing data are handled. Completers-only or inadequately handled missing data may affect the originally randomization-based composition of the conditions, because, for example, deteriorating participants may tend to drop out (Rozental et al., 2014). This may in turn artificially inflate the effect of the intervention (Andrade, 2022). Moreover, it may be expected that studies willing to provide IPD are methodologically rigorous, and such studies tend to report lower effect sizes (Cuijpers et al., 2023b). Indeed, more studies among the ones that provided data for our IPDMA (*k* = 3) than those that did not (*k* = 1) were evaluated as having low risk of bias in our conventional MA risk of bias evaluation.

We identified baseline depressive symptoms and comorbid anxiety as prognostic factors but not effect modifiers in internet-based interventions for perinatal depression. Baseline depressive symptoms have been identified as a prognostic factor in general adult populations (Karyotaki et al., 2021). Moreover, they were listed as an effect modifier in the comparison of interventions to controls in several previous IPDMAs focused on psychological interventions for depression (Bower et al., 2013; Karyotaki et al., 2018a; Kuyken et al., 2016; Reins et al., 2021), and in IPD-NMAs comparing guided and self-guided internet-based interventions to controls (Karyotaki et al., 2021), or cognitive behavioral analysis system of psychotherapy (CBASP) for persistent depression compared to combined treatment or antidepressants only (Furukawa et al., 2018). Comorbid anxiety was not found to be a prognostic factor or effect modifier in psychological interventions for depression in some IPDMAs (Karyotaki et al., 2018a; Karyotaki et al., 2017), but it was an effect modifier in CBASP in comparison to antidepressants and combined treatment in persistent depression (Furukawa et al., 2018). Similarly, we did not find any prognostic factors of adherence to the interventions, while previous studies reported age, education, employment or anxiety (Karyotaki et al., 2015; Tong et al., 2026). These associations may, however, appear in certain populations and for certain types of treatments, as opposed to the very specific population with perinatal depression.

The current study is the first one to provide estimates of outcomes of internet-based interventions for perinatal depression, including reduction in depressive symptoms, response, remission, adherence, deterioration, prognostic factors and effect modifiers, which have not been reported earlier. In addition, the methodology applied in the current study is rigorous and provides robust estimates (Karyotaki et al., 2022), which were calculated using state-of-the-art methods for statistical synthesis (Harrer et al., 2021; Harrer et al., 2023), allowing for the application of these methods in a standardized manner across the included studies (Cuijpers et al., 2022). By collecting primary data, we increased statistical power in comparison to RCTs, and were able to estimate not only study- but also participant-level characteristics, which is an advantage in comparison to conventional MAs (Karyotaki et al., 2018b).

The results of the current study must be, however, considered in the light of their limitations. First, we could not assess all prognostic factors, effect modifiers, and outcomes that we aimed to assess. None of the identified studies compared a blended intervention arm to a control condition, none compared guided to self-guided interventions directly, and only one study compared blended to guided intervention. The number of studies which provided data was also too small to conduct sensitivity analyses separately for guided and self-guided interventions. There were also many potential outcomes, prognostic factors, and effect modifiers, which have been studied rarely or not at all (for a full overview, see Appendix B). Second, the results show considerable uncertainty, possibly related to the large variability among studies in terms of intervention timing, guidance, recruitment, control conditions, and region: moderate to high heterogeneity, broad prediction interval, indication of publication bias, and differing results when only low risk of bias studies were included. Moreover, 53% of the studies included in the conventional MA were at high risk of bias. We found very little evidence that any of these variables were associated with different effect sizes, but more exploration is necessary. Third, deterioration was a very rare outcome. Although this analysis was handled by state-of-the-art methods to assess sparse data (Klüppelberg et al., 2014), caution must be taken while interpreting results of respective analyses, because they are based on low levels of information. Fourth, only 59% of included studies provided their data, which is a lower percentage than other IPDMAs on depression (Karyotaki et al., 2022; Karyotaki et al., 2018b). However, we did not find any evidence of a difference in effects reported in the publications of the studies which did and did not provide data, and between our conventional and IPDMA. Including additional studies might thus not change the current results, even though data availability bias cannot be fully ruled out. Fifth, most studies focused on CBT. The current results might therefore not be generalizable to other therapeutic approaches. Sixth, adherence was defined as the percentage of completed modules, but the number of modules, their length and intensity of content likely differed across the studies. Although this was the most practical manner to standardize adherence, the treatment dosage differed among participants considered to show the same adherence to the treatment. Future research should address engagement with treatments using other definitions. Finally, prognostic factors identified in the current study provide only correlational evidence and cannot be therefore considered a causal factor of post-treatment symptoms or adherence.

Despite these limitations, the current IPDMA provides support to the provision of internet-based interventions to new and expectant mothers who experience symptoms of perinatal depression. Within the limitations of the tested variables, it seems that no specific subgroup particularly benefits from these interventions, deteriorates when receiving them, or tends to drop out. Therefore, these interventions may be offered to patients regardless of their age, education, relationship status, employment, baseline depressive or anxiety symptoms, comorbid anxiety, previous treatment, number of children, or ethnic minority status, if this is in line with their preferences. Yet, effects are small, and other, untested characteristics, may modify them.

The results of the current study may further lead us in which directions optimization of treatments may aim. Participants with higher depressive symptoms and comorbid anxiety score higher also at later time points, regardless of intervention. This may suggest that such participants could benefit from more intensive treatments, perhaps in terms of more frequent modules, which have been suggested to lead to better outcomes (Ciharova et al., 2024), rather than in terms of a richer content or more sessions, as more severe symptoms may hinder concentration and engagement (Franco et al., 2024) and mothers in the perinatal period may struggle to find time to engage with long, intensive interventions, similarly to parents in general (Gilson et al., 2018). After all, it seems that the current clinical effects are observed regardless of suboptimal adherence described in the current study. They should also be more carefully followed by a clinician.

Future research is needed into effect modifiers of long-term effects of internet-based interventions for perinatal depression. A direct comparison of guided, self-guided, blended and face-to-face treatments, for example by means of an IPD-NMA, is further needed, so that we can distinguish between participants who require traditional sessions with a therapist, and for whom internet-based approaches suffice or are preferred. For this research direction to take place, it would be desirable for RCTs to start collecting data on topics that may be relevant in the perinatal period, but are currently often not being assessed, such as breastfeeding satisfaction, pregnancy or childbirth complications, (mental) health outcomes in the infant and the partner, or the relationship between the mother and the infant, as well as factors influencing adherence to digital interventions, such as internet access, digital literacy, or previous experience with digital interventions, among others. Alternatively, data registered in routine care settings, such as hospitals, should become available for similar analyses. Collecting such data would enable further IPDMAs to assess whether these variables are associated with effects of interventions for perinatal mental health. It is also unclear whether partner involvement increases the effectiveness of the intervention. Moreover, current RCTs often suffer from risk of and publication bias, especially unclear reporting of methods, lack of preregistration, and insufficient handling of missing data, more methodological rigor is thus needed. Finally, we have seen that analysis of deterioration data is challenging, especially due to deterioration being a very rare event. While this may be a positive piece of news, large RCTs are necessary to answer the question who tends to deteriorate in internet-based interventions more often, and how we can prevent it. Alternatively, more lenient definitions of deterioration may be used (Wise, 2004).

To conclude, the current study showed that internet-based interventions for perinatal depression are effective for both depression and anxiety, although the effects are modest. We did not identify robust evidence for modifiers of effects of the interventions, or prognostic factors of decreased adherence among the tested variables, namely age, education, relationship status, employment, baseline depressive or anxiety symptoms, comorbid anxiety, previous treatment, number of children, or ethnic minority status. Therefore, internet-based interventions may be offered to new and expectant mothers experiencing depressive symptoms regardless of these characteristics, if they wish to follow them. The current results are restricted by lack of research into long-term effects and potential prognostic factors and effect modifiers so far not studied, a limitation future RCTs should focus on.

## Declaration of Generative AI and AI-assisted technologies in the writing process

During the preparation of this work the authors used ChatGPT-5.2 in order to consult it when creating the R code for the current analysis. After using this tool, the authors reviewed and edited the content as needed and take full responsibility for the content of the published article.

## Funding sources

This project was supported by the 10.13039/501100000925National Health and Medical Research Council's (NHMRC) Centre of Research Excellence (CRE) scheme (STePPS CRE: Science Translation for e-Psychological Perinatal Supports). PF's contribution to this work was supported by the Chilean National Agency for 10.13039/100006190Research and Development (ANID) through a Fondecyt de Postdoctorado grant (Folio No. 3250136). The funders had no involvement in the study design, data collection, analysis, interpretation, or the writing of the manuscript.

## Declaration of competing interest

The authors have no interests to declare.

## Data Availability

We published the protocol for this study in Open Science Framework (https://osf.io/98tms) and provided the statistical analysis plan in the Supplementary materials. We will make the data dictionary available upon the publication of the current study. We may share deidentified participant data if the authors of the primary study agree, and if the receiver signs any required data sharing agreements. Please, direct requests regarding data to MC at m.ciharova@vu.nl.
