# Supplementary material for: Modifiers of effectiveness and deterioration, and prognostic factors of adherence in internet-based interventions for treatment of perinatal depression: A systematic review and individual participant data meta-analysis
Source: Internet Interv. 2026 Sep 3;46:100993. doi: 10.1016/j.invent.2026.100993 (PMC13571255; doi:10.1016/j.invent.2026.100993)
Supplement: Supplementary file 1 [file mmc1.docx]

Supplementary Materials

Modifiers of Effectiveness and Deterioration, and Prognostic Factors of Adherence in Internet-Based Interventions for Treatment of Perinatal Depression: A Systematic Review and Individual Participant Data Meta-analysis

Table of Contents

[Appendix A. PRISMA Checklist	2](#_Toc233532298)

[Appendix B. Deviations from the Registered Protocol	6](#_Toc233532299)

[Appendix C. Search strings	7](#_Toc233532300)

[PubMed	7](#_Toc233532301)

[Embase	10](#_Toc233532302)

[APA PsycInfo	14](#_Toc233532303)

[Appendix D. Study-level Variables Extracted from Published Studies	22](#_Toc233532304)

[Appendix E. Requested Individual Participant Data Variables	23](#_Toc233532305)

[Appendix F. Conventional Meta-analysis – Analysis Details	24](#_Toc233532306)

[Appendix G. Multiple Imputation	25](#_Toc233532307)

[Details of the Analysis	25](#_Toc233532308)

[Visualization of Imputation Matrices per Study	25](#_Toc233532309)

[Appendix H. IPDMA – Analysis Detail and Sensitivity Analyses	30](#_Toc233532310)

[Appendix I. PRISMA IPD Flow Diagram	31](#_Toc233532311)

[Appendix J. References of the Studies Included in the Conventional Meta-Analysis	32](#_Toc233532312)

[Appendix K. References of the Studies Included in the Individual Participant Data Meta-Analysis	34](#_Toc233532313)

[Appendix L. Risk of Bias of Studies Included in the IPDMA	35](#_Toc233532314)

[Risk of bias traffic light plot	35](#_Toc233532315)

[Risk of bias summary plot	35](#_Toc233532316)

[Appendix M. Risk of Bias of Studies Included in the Conventional Meta-analysis	36](#_Toc233532317)

[Risk of bias traffic light plot	36](#_Toc233532318)

[Risk of bias summary plot	36](#_Toc233532319)

[Appendix N. Results of Conventional Meta-analysis	37](#_Toc233532320)

[Conventional meta-analysis with sensitivity analyses	37](#_Toc233532321)

[Multivariate meta-regression analysis assessing the impact of IPD provision	37](#_Toc233532322)

[Forest plot	38](#_Toc233532323)

[Funnel plot	38](#_Toc233532324)

[Comparison of studies which did and did not provide individual participant data	39](#_Toc233532325)

[Appendix O. Participant Characteristics (Completers of Questionnaires Only)	40](#_Toc233532326)

[Appendix P. Forest and funnel plots for the Individual Participant Data Meta-analysis	41](#_Toc233532327)

[Forest plot	41](#_Toc233532328)

[Funnel plot	41](#_Toc233532329)

[Appendix Q. The Strength of Evidence of Our Main Findings as Evaluated Based on the GRADE Assessment	42](#_Toc233532330)

[Appendix R. Sensitivity Analyses – Effectiveness of Internet-Based Interventions for Perinatal Depression	43](#_Toc233532331)

[Appendix S. Sensitivity Analyses and Additional Information for the Adherence Analysis	44](#_Toc233532332)

[Prognostic Factors of Adherence to Internet-Based Interventions for Perinatal Depression Analyzed with Poisson Regression	44](#_Toc233532333)

[Prognostic Factors of Adherence to Internet-Based Interventions for Perinatal Depression Analyzed Separately for Guided Interventions (Beta Regression)	44](#_Toc233532334)

[References (Supplementary materials)	45](#_Toc233532335)

# **Appendix A. PRISMA Checklist**

| **PRISMA-IPD**  **Section/topic** | **Item No** | **Checklist item** | **Reported on page** |
| --- | --- | --- | --- |
| **Title** | | | |
| Title | 1 | Identify the report as a systematic review and meta-analysis of individual participant data. | 1 |
| **Abstract** | | | |
| Structured summary | 2 | Provide a structured summary including as applicable: | 3 |
|  |  | **Background**: state research question and main objectives, with information on participants, interventions, comparators and outcomes. |  |
|  |  | **Methods**: report eligibility criteria; data sources including dates of last bibliographic search or elicitation, noting that IPD were sought; methods of assessing risk of bias. |  |
|  |  | **Results**: provide number and type of studies and participants identified and number (%) obtained; summary effect estimates for main outcomes (benefits and harms) with confidence intervals and measures of statistical heterogeneity. Describe the direction and size of summary effects in terms meaningful to those who would put findings into practice. |  |
|  |  | **Discussion:** state main strengths and limitations of the evidence, general interpretation of the results and any important implications. |  |
|  |  | **Other:** report primary funding source, registration number and registry name for the systematic review and IPD meta-analysis. |  |
| **Introduction** | | | |
| Rationale | 3 | Describe the rationale for the review in the context of what is already known. | 4-5 |
| Objectives | 4 | Provide an explicit statement of the questions being addressed with reference, as applicable, to participants, interventions, comparisons, outcomes and study design (PICOS). Include any hypotheses that relate to particular types of participant-level subgroups. | 5-6 |
| **Methods** | | | |
| Protocol and registration | 5 | Indicate if a protocol exists and where it can be accessed. If available, provide registration information including registration number and registry name. Provide publication details, if applicable. | 6 |
| Eligibility criteria | 6 | Specify inclusion and exclusion criteria including those relating to participants, interventions, comparisons, outcomes, study design and characteristics (e.g. years when conducted, required minimum follow-up). Note whether these were applied at the study or individual level i.e. whether eligible participants were included (and ineligible participants excluded) from a study that included a wider population than specified by the review inclusion criteria. The rationale for criteria should be stated. | 6-7 |
| Identifying studies - information sources | 7 | Describe all methods of identifying published and unpublished studies including, as applicable: which bibliographic databases were searched with dates of coverage; details of any hand searching including of conference proceedings; use of study registers and agency or company databases; contact with the original research team and experts in the field; open adverts and surveys. Give the date of last search or elicitation. | 6 |
| Identifying studies - search | 8 | Present the full electronic search strategy for at least one database, including any limits used, such that it could be repeated. | Appendix C |
| Study selection processes | 9 | State the process for determining which studies were eligible for inclusion. | 6 |
| Data collection processes | 10 | Describe how IPD were requested, collected and managed, including any processes for querying and confirming data with investigators. If IPD were not sought from any eligible study, the reason for this should be stated (for each such study). | 7 |
|  |  | If applicable, describe how any studies for which IPD were not available were dealt with. This should include whether, how and what aggregate data were sought or extracted from study reports and publications (such as extracting data independently in duplicate) and any processes for obtaining and confirming these data with investigators. |  |
| Data items | 11 | Describe how the information and variables to be collected were chosen. List and define all study level and participant level data that were sought, including baseline and follow-up information. If applicable, describe methods of standardising or translating variables within the IPD datasets to ensure common scales or measurements across studies. | 7  Appendices D and E |
| IPD integrity | A1 | Describe what aspects of IPD were subject to data checking (such as sequence generation, data consistency and completeness, baseline imbalance) and how this was done. | 7 |
| Risk of bias assessment in individual studies. | 12 | Describe methods used to assess risk of bias in the individual studies and whether this was applied separately for each outcome. If applicable, describe how findings of IPD checking were used to inform the assessment. Report if and how risk of bias assessment was used in any data synthesis. | 7 |
| Specification of outcomes and effect measures | 13 | State all treatment comparisons of interests. State all outcomes addressed and define them in detail. State whether they were pre-specified for the review and, if applicable, whether they were primary/main or secondary/additional outcomes. Give the principal measures of effect (such as risk ratio, hazard ratio, difference in means) used for each outcome. | 8-10 |
| Synthesis methods | 14 | Describe the meta-analysis methods used to synthesise IPD. Specify any statistical methods and models used. Issues should include (but are not restricted to):   - Use of a one-stage or two-stage approach. - How effect estimates were generated separately within each study and combined across studies (where applicable). - Specification of one-stage models (where applicable) including how clustering of patients within studies was accounted for. - Use of fixed or random effects models and any other model assumptions, such as proportional hazards. - How (summary) survival curves were generated (where applicable). - Methods for quantifying statistical heterogeneity (such as I^2^ and τ^2^). - How studies providing IPD and not providing IPD were analysed together (where applicable). - How missing data within the IPD were dealt with (where applicable). | 8-10 |
| Exploration of variation in effects | A2 | If applicable, describe any methods used to explore variation in effects by study or participant level characteristics (such as estimation of interactions between effect and covariates). State all participant-level characteristics that were analysed as potential effect modifiers, and whether these were pre-specified. | 9-10 |
| Risk of bias across studies | 15 | Specify any assessment of risk of bias relating to the accumulated body of evidence, including any pertaining to not obtaining IPD for particular studies, outcomes or other variables. | Appendices F and H |
| Additional analyses | 16 | Describe methods of any additional analyses, including sensitivity analyses. State which of these were pre-specified. | Appendices B, F and H |
| **Results** | | | |
| Study selection and IPD obtained | 17 | Give numbers of studies screened, assessed for eligibility, and included in the systematic review with reasons for exclusions at each stage. Indicate the number of studies and participants for which IPD were sought and for which IPD were obtained. For those studies where IPD were not available, give the numbers of studies and participants for which aggregate data were available. Report reasons for non-availability of IPD. Include a flow diagram. | 10, Appendix I |
| Study characteristics | 18 | For each study, present information on key study and participant characteristics (such as description of interventions, numbers of participants, demographic data, unavailability of outcomes, funding source, and if applicable duration of follow-up). Provide (main) citations for each study. Where applicable, also report similar study characteristics for any studies not providing IPD. | Tables 1 and 2, Appendices J and K |
| IPD integrity | A3 | Report any important issues identified in checking IPD or state that there were none. | 11 |
| Risk of bias within studies | 19 | Present data on risk of bias assessments. If applicable, describe whether data checking led to the up-weighting or down-weighting of these assessments. Consider how any potential bias impacts on the robustness of meta-analysis conclusions. | 11 |
| Results of individual studies | 20 | For each comparison and for each main outcome (benefit or harm), for each individual study report the number of eligible participants for which data were obtained and show simple summary data for each intervention group (including, where applicable, the number of events), effect estimates and confidence intervals. These may be tabulated or included on a forest plot. | Table 1 |
| Results of syntheses | 21 | Present summary effects for each meta-analysis undertaken, including confidence intervals and measures of statistical heterogeneity. State whether the analysis was pre-specified, and report the numbers of studies and participants and, where applicable, the number of events on which it is based. | Tables 3-5, Appendices B, O, P, Q, R, S |
|  |  | When exploring variation in effects due to patient or study characteristics, present summary interaction estimates for each characteristic examined, including confidence intervals and measures of statistical heterogeneity. State whether the analysis was pre-specified. State whether any interaction is consistent across trials. |  |
|  |  | Provide a description of the direction and size of effect in terms meaningful to those who would put findings into practice. |  |
| Risk of bias across studies | 22 | Present results of any assessment of risk of bias relating to the accumulated body of evidence, including any pertaining to the availability and representativeness of available studies, outcomes or other variables. | Appendices N and R |
| Additional analyses | 23 | Give results of any additional analyses (e.g. sensitivity analyses). If applicable, this should also include any analyses that incorporate aggregate data for studies that do not have IPD. If applicable, summarise the main meta-analysis results following the inclusion or exclusion of studies for which IPD were not available. | Appendices R and S |
| **Discussion** | | | |
| Summary of evidence | 24 | Summarise the main findings, including the strength of evidence for each main outcome. | 13 |
| Strengths and limitations | 25 | Discuss any important strengths and limitations of the evidence including the benefits of access to IPD and any limitations arising from IPD that were not available. | 14-15 |
| Conclusions | 26 | Provide a general interpretation of the findings in the context of other evidence. | 13-14 |
| Implications | A4 | Consider relevance to key groups (such as policy makers, service providers and service users). Consider implications for future research. | 15-16 |
| **Funding** | | | |
| Funding | 27 | Describe sources of funding and other support (such as supply of IPD), and the role in the systematic review of those providing such support. | 17 |

# **Appendix B. Deviations from the Registered Protocol**

In the conventional meta-analysis, we had to avoid a subgroup analysis assessing the differences in effects related to the type of measure used (i.e., self-report vs clinician-rated), because all studies used self-reported measures. Furthermore, we did not assess the differences between high- and low- and middle-income countries, because these groups almost fully overlaped with another subgroup analysis which we conducted, namely to assess the differences between western and non-western countries. We chose the latter distinction, because the former rended only four studies in the subgroup of low- and middle-income countries, while the latter revealed six, meaning that more reliable estimates could be delivered.

In the IPDMA, the following prognostic factors and effect modifiers could not be tested, as less than five included studies provided data for them: episode duration, suicidal ideation, presence of domestic violence, comorbid mental disorders (other than anxiety), comorbid physical disorder, pregnancy complications, (absence of) breatfeeding, cultural background (other than minority status), mother-infant and mother-partner relationship, treatment expectancy and burden, presence of interventions delivered to the infant or the partner during the treatment period, health outcomes in the infant and the partner. We also could not test the effectiveness of interventions aimed primarily on the infant and the partner, or the effectiveness of the interventions delivered to the mother on health outcomes in the infant and the partner. In addition, we could not test blended interventions, as only one of the included studies was in this format and compared its effectiveness only to a guided intervention. Finally, we did not assess follow-up (i.e., long-term) outcomes, because only three studies collected such data, and the timings of the follow-up measurements in these studies differed.

In the analysis of prognostic factors and effect modifiers of effectiveness, we could not assess relationship status as an effect modifier in the studies by Forsell and Milgrom, 2021, due to lack of variation. Similarly, minority status as an effect modifier could not be tested in the study by Franco and Pugh.

In the analysis of prognostic factors of adherence, some studies had to be excluded from individual binary analyses due to sparse data in some levels of categorical predictors, or no variation in continuous predictors. For the main analysis conducted using beta regression, it was relationship and minority status for Franco, education and baseline anxiety for Milgrom, 2016, and relationship, employment, minority status and number of children for Milgrom, 2021. For Poisson regression, it was relationship and minority status for Franco and relationship status for Milgrom, 2021.

# **Appendix C. Search strings**

## PubMed

\# Depression

"Depression"[Mesh] OR "Depressive Disorder"[Mesh] OR “depress*”[tiab] OR “dysthymi*”[tiab] OR “mood disorder*”[tiab] OR “affective disorder*”[tiab] OR “dysphoric disorder*”[tiab]

#Anxiety

“Anxiety disorders” [Mesh] OR "Anxiety"[Mesh] OR "Fear"[Mesh] OR "shyness"[MeSH Terms] OR shyness[tiab] OR shy[tiab] OR “anxiet*”[tiab] OR “agoraphobi*”[tiab] OR “panic”[tiab] OR “social phobi*”[tiab] OR “phobi*”[tiab] OR “obsessive-compulsive”[tiab] OR “neurotic disorder*”[tiab] OR “hoarding*”[tiab] OR “OCD”[tiab] OR “neurotic anxiet*”[tiab] OR “anxious*”[tiab] OR “emotional disorder*”[tiab] OR fear[tiab] OR worry[tiab] OR worrying[tiab] OR worries[tiab] OR GAD[tiab] OR Arachnophobia[tiab] OR Ophidiophobia[tiab] OR Acrophobia[tiab] OR Cynophobia[tiab] OR Claustrophobia[tiab] OR Mysophobia[tiab] OR Aerophobia[tiab] OR Trypophobia[tiab] OR Carcinophobia[tiab] OR Thanatophobia[tiab] OR Glossophobia[tiab] OR Monophobia[tiab] OR Ornithophobia[tiab] OR Alektorophobia[tiab] OR Trypanophobia[tiab] OR Anthropophobia[tiab] OR Aquaphobia[tiab] OR Autophobia[tiab] OR Hemophobia[tiab] OR Xenophobia[tiab] OR Ailurophobia[tiab] OR Nyctophobia[tiab] OR Phobophobia[tiab] OR Philophobia[tiab] OR Triskaidekaphobia[tiab] OR Emetophobia[tiab] OR Entomophobia[tiab] OR Zoophobia[tiab] OR Scelerophobia[tiab] OR Cibophobia[tiab] OR Tokophobia[tiab] OR Pseudodysphagia[tiab] OR Gerascophobia[tiab] OR Technophobia[tiab] OR Ergophobia[tiab] OR Coulrophobia [tiab] OR Photophobia[tiab] OR Numerophobia[tiab] OR Taphophobia[tiab]

\# Psychological interventions

“Psychotherapy”[Mesh] OR “Counseling”[Mesh] OR psychotherap*[Tiab] OR cbt[Tiab] OR counselling[Tiab] OR counseling[Tiab] OR “Eye Movement Desensitization Reprocessing”[tiab] OR “Eye Movement Desensitization and Reprocessing”[tiab] OR “Eye Movement Desensitisation Reprocessing”[tiab] OR “Eye Movement Desensitisation and Reprocessing”[tiab] OR EMDR[tiab] OR “Bibliotherap*”[tiab] OR mindfulness[Tiab] OR “Autogenic Training”[tiab] OR Logotherap*[tiab] OR "cognitive restructuring"[Tiab] OR "self-control training*"[Tiab] OR "assertiveness training"[Tiab] OR ((“therapy”[SubHeading] OR therap*[Tiab] OR “Therapeutics”[Mesh] OR treatment*[Tiab] OR intervention*[tiab]) AND (“brief psychodynamic”[Tiab] OR “short psychodynamic”[tiab] OR "problem-solving"[Tiab] OR "compassion-focused"[Tiab] OR "compassion-focussed"[Tiab] OR “compassion-based”[tiab] OR constructivist*[Tiab] OR metacognitive[tiab] OR “meta-cognitive”[Tiab] OR "solution-focused"[Tiab] OR "solution-focussed"[Tiab] OR "self-control”[Tiab] OR psychosocial[tiab] OR “peer support“[tiab] OR “task-shifted”[tiab] OR Relaxation[tiab] OR “dialectical behavior”[tiab] OR “emotion-focused”[tiab] OR narrative[tiab] OR “person-centred”[tiab] OR “person-centered”[tiab] OR “Narrative”[tiab] OR “meaning-centered”[tiab] OR “humanistic”[tiab] OR “client-centered”[tiab] OR “meaning-centred”[tiab] OR “client-centred”[tiab] OR “Rogerian”[tiab] OR “Nondirective”[tiab] OR “Non-directive”[tiab] OR “Supportive”[tiab] OR “Life review”[tiab] OR "acceptance and commitment"[Tiab] OR (“schema”[tiab] AND brief[tiab]) OR (“gestalt”[tiab] AND brief[tiab]))) OR "behavior therap*"[Tiab] OR "behaviors therap*"[Tiab] OR "behavioral therap*"[Tiab] OR "behaviour therap*"[Tiab] OR "behaviours therap*"[Tiab] OR "behavioural therap*"[Tiab] OR "cognition therap*"[Tiab] OR “cognitive therap*”[tiab] OR "behavior treatment*"[Tiab] OR "behaviors treatment*"[Tiab] OR "behavioral treatment*"[Tiab] OR "behaviour treatment*"[Tiab] OR "behaviours treatment*"[Tiab] OR "behavioural treatment*"[Tiab] OR "cognition treatment*"[Tiab] OR “cognitive treatment*”[tiab] OR "behavior intervention*"[Tiab] OR "behaviors intervention*"[Tiab] OR "behavioral intervention*"[Tiab] OR "behaviour intervention*"[Tiab] OR "behaviours intervention*"[Tiab] OR "behavioural intervention*"[Tiab] OR "cognition intervention*"[Tiab] OR “cognitive intervention*”[tiab] OR "behavior activation*"[Tiab] OR "behaviors activation*"[Tiab] OR "behavioral activation*"[Tiab] OR "behaviour activation*"[Tiab] OR "behaviours activation*"[Tiab] OR "behavioural activation*"[Tiab] OR exposure[tiab]

\# Internet-based

"Telemedicine"[Mesh] OR "Mobile Applications"[Mesh] OR "Social Media"[Mesh] OR "Therapy, Computer-Assisted"[Mesh:NoExp] OR "Drug Therapy, Computer-Assisted"[Mesh:NoExp] OR "Telecommunications"[Mesh:NoExp] OR "Electronic Mail"[Mesh] OR "Videoconferencing"[Mesh] OR "Cell Phone"[Mesh] OR "Distance Counseling"[Mesh] OR “Wearable Electronic Devices”[Mesh] OR telehealth[tiab] OR “tele-health”[tiab] OR telepsychology[tiab] OR “tele-psychology”[tiab] OR telepsychiatry[tiab] OR “tele-psychiatry”[tiab] OR “tele-therap*”[tiab] OR teletherap*[tiab] OR “tele-medicine”[tiab] OR telemedicine[tiab] OR telecare[tiab] OR “tele-care”[tiab] OR telecommunicat*[tiab] OR “tele-communicat*”[tiab] OR teleconference*[tiab] OR “tele-conferenc*”[tiab] OR videoconferenc*[tiab] OR “video-conferenc*”[tiab] OR computer*[tiab] OR electronic*[tiab] OR digital*[tiab] OR ehealth[tiab] OR “e-health”[tiab] OR “e-treat*”[tiab] OR “e-therap*”[tiab] OR mhealth[tiab] OR “m-health”[tiab] OR “internet-based*”[tiab] OR “internet treat*”[tiab] OR “internet intervention*”[tiab] OR “internet counsel*”[tiab] OR “distance counsel*”[tiab] OR “web-based*”[tiab] OR cybercounsel*[tiab] OR “cyber-counsel*”[tiab] OR “online treat*”[tiab] OR “online therap*”[tiab] OR “online intervention*”[tiab] OR “online prevention*”[tiab] OR “online counsel*”[tiab] OR “text-messag*”[tiab] OR textmessag*[tiab] OR SMS[tiab] OR texting*[tiab] OR “short message service*”[tiab] OR mobile*[tiab] OR smartphone*[tiab] OR “cell-phone*”[tiab] OR cellphone*[tiab] OR “cellular phone*”[tiab] OR blended*[tiab] OR “software app*”[tiab] OR “handheld device*”[tiab] OR “hand held device*”[tiab] OR iPad*[tiab] OR iPhone*[tiab] OR email*[tiab] OR “e-mail*”[tiab] OR sensor*[tiab] OR wearable*[tiab] OR “social media*”[tiab] OR “social network*”[tiab] OR “e-counsel*”[tiab] OR ecounsel*[tiab] OR palmtop*[tiab] OR telephone*[tiab] OR WhatsApp[tiab] OR Twitter[tiab] OR Facebook[tiab] OR Instagram[tiab] OR forum[tiab] OR chat*[tiab] OR “virtual reality*”[tiab] OR avatar*[tiab] OR “Conversational agent*”[tiab] OR “virtual coach”[tiab] OR “virtual agent*”[tiab] OR “embodied agent*”[tiab] OR “relational agent*”[tiab] OR “interactive agent*”[tiab] OR “virtual character*”[tiab] OR “virtual human*”[tiab] OR “virtual assistant*”[tiab] OR VR[tiab] OR “serious game*”[tiab] OR “serious gaming”[tiab] OR gamification[tiab]

\# Trials/ SR/ Meta-analysis

"Meta-Analysis" [Publication Type] OR "Meta-Analysis as Topic"[Mesh] OR metaanaly*[tiab] OR meta-analy*[tiab] or metanaly*[tiab] OR "Systematic Review" [Publication Type] OR systematic[sb] OR meta-analysis[Filter] OR systematicreview[Filter] OR "Cochrane Database Syst Rev"[Journal] or prisma[tiab] OR “preferred reporting items”[tiab] OR prospero[tiab] OR ((systemati*[ti] OR umbrella[ti] OR “structured literature”[ti]) AND (review[ti] OR overview[ti])) OR “systematic review”[tiab] OR “umbrella review”[tiab] OR “structured literature review”[tiab] OR “systematic qualitative review”[tiab] OR “systematic quantitative review”[tiab] OR “systematic search and review”[tiab] OR “systematized review”[tiab] OR “systematised review”[tiab] OR “systemic review”[tiab] OR “systematic literature review”[tiab] OR “systematic integrative literature review”[tiab] OR “systematically review”[tiab] OR “scoping literature review”[tiab] OR “scoping review”[tiab] OR “systematic critical review”[tiab] OR “systematic integrative review”[tiab] OR “systematic evidence review”[tiab] OR “systematic integrative literature review”[tiab] OR “systematic mixed studies review”[tiab] OR “systematized literature review”[tiab] OR “systematic overview”[tiab] OR “Systematic narrative review”[tiab] OR “narrative review”[tiab] OR metasynthes*[tiab] OR meta-synthes*[tiab] OR “Randomized Controlled Trial”[pt] OR “Randomized Controlled Trials as Topic”[Mesh] OR “Random allocation” [Mesh] OR “Double-blind method”[Mesh] OR “Single-blind method”[Mesh] OR random[tiab] OR randomly[tiab] OR randomised[tiab] OR randomized[tiab] OR randomising[tiab] OR randomizing [tiab] OR ((singl*[tiab] OR doubl*[tiab] OR trebl*[tiab] OR tripl*[tiab]) AND (mask*[tiab] OR blind*[tiab] OR dumm*[tiab])) OR RCT[tiab] OR "Clinical Trials as Topic"[Mesh] OR “Clinical Trial”[pt] OR “clinical trial*”[tiab] OR “Controlled Clinical Trial”[pt] OR “controlled trial*”[tiab]

\# Publication type

NOT ("Comment" [Publication Type] OR "Letter" [Publication Type] OR "Editorial" [Publication Type] OR (("Animals"[Mesh] OR "Models, Animal"[Mesh]) NOT "Humans"[Mesh]))

\# Timeframe

After 2000

## Embase

\# Depression

'depression'/exp OR ‘depress*’:ab,ti,kw OR ‘dysthymi*’:ab,ti,kw OR ‘mood disorder*’:ab,ti,kw OR ‘affective disorder*’:ab,ti,kw OR ‘dysphoric disorder*’:ab,ti,kw

#Anxiety

'anxiety disorder'/exp OR 'anxiety'/exp OR 'fear'/de OR 'shyness'/exp OR shyness:ab,ti,kw OR shy:ab,ti,kw OR ‘anxiet*’:ab,ti,kw OR ‘agoraphobi*’:ab,ti,kw OR ‘panic’:ab,ti,kw OR ‘social phobi*’:ab,ti,kw OR ‘phobi*’:ab,ti,kw OR ‘obsessive-compulsive’:ab,ti,kw OR ‘neurotic disorder*’:ab,ti,kw OR ‘hoarding*’:ab,ti,kw OR ‘OCD’:ab,ti,kw OR ‘neurotic anxiet*’:ab,ti,kw OR ‘anxious*’:ab,ti,kw OR ‘emotional disorder*’:ab,ti,kw OR fear:ab,ti,kw OR worry:ab,ti,kw OR worrying:ab,ti,kw OR worries:ab,ti,kw OR GAD:ab,ti,kw OR Arachnophobia:ab,ti,kw OR Ophidiophobia:ab,ti,kw OR Acrophobia:ab,ti,kw OR Cynophobia:ab,ti,kw OR Claustrophobia:ab,ti,kw OR Mysophobia:ab,ti,kw OR Aerophobia:ab,ti,kw OR Trypophobia:ab,ti,kw OR Carcinophobia:ab,ti,kw OR Thanatophobia:ab,ti,kw OR Glossophobia:ab,ti,kw OR Monophobia:ab,ti,kw OR Ornithophobia:ab,ti,kw OR Alektorophobia:ab,ti,kw OR Trypanophobia:ab,ti,kw OR Anthropophobia:ab,ti,kw OR Aquaphobia:ab,ti,kw OR Autophobia:ab,ti,kw OR Hemophobia:ab,ti,kw OR Xenophobia:ab,ti,kw OR Ailurophobia:ab,ti,kw OR Nyctophobia:ab,ti,kw OR Phobophobia:ab,ti,kw OR Philophobia:ab,ti,kw OR Triskaidekaphobia:ab,ti,kw OR Emetophobia:ab,ti,kw OR Entomophobia:ab,ti,kw OR Zoophobia:ab,ti,kw OR Scelerophobia:ab,ti,kw OR Cibophobia:ab,ti,kw OR Tokophobia:ab,ti,kw OR Pseudodysphagia:ab,ti,kw OR Gerascophobia:ab,ti,kw OR Technophobia:ab,ti,kw OR Ergophobia:ab,ti,kw OR Coulrophobia:ab,ti,kw OR Photophobia:ab,ti,kw OR Numerophobia:ab,ti,kw OR Taphophobia:ab,ti,kw

\# Psychotherapy

'psychotherapy'/exp OR 'counseling'/exp OR psychotherap*:ab,ti,kw OR cbt:ab,ti,kw OR counselling:ab,ti,kw OR counseling:ab,ti,kw OR ‘Eye Movement Desensitization Reprocessing’:ab,ti,kw OR ‘Eye Movement Desensitization and Reprocessing’:ab,ti,kw OR ‘Eye Movement Desensitisation Reprocessing’:ab,ti,kw OR ‘Eye Movement Desensitisation and Reprocessing’:ab,ti,kw OR EMDR:ab,ti,kw OR ‘Bibliotherap*’:ab,ti,kw OR mindfulness:ab,ti,kw OR ‘Autogenic Training’:ab,ti,kw OR Logotherap*:ab,ti,kw OR ‘cognitive restructuring’:ab,ti,kw OR ‘self-control training*’:ab,ti,kw OR ‘assertiveness training’:ab,ti,kw OR (('therapy'/lnk OR 'therapy'/exp OR therap*:ab,ti,kw OR treatment*:ab,ti,kw OR intervention*:ab,ti,kw) AND (‘brief psychodynamic’:ab,ti,kw OR ‘short psychodynamic’:ab,ti,kw OR ‘problem-solving’:ab,ti,kw OR ‘compassion-focused’:ab,ti,kw OR ‘compassion-focussed’:ab,ti,kw OR ‘compassion-based’:ab,ti,kw OR constructivist*:ab,ti,kw OR metacognitive:ab,ti,kw OR ‘meta-cognitive’:ab,ti,kw OR ‘solution-focused’:ab,ti,kw OR ‘solution-focussed’:ab,ti,kw OR ‘self-control’:ab,ti,kw OR psychosocial:ab,ti,kw OR ‘peer support’:ab,ti,kw OR ‘task-shifted’:ab,ti,kw OR Relaxation:ab,ti,kw OR ‘dialectical behavior’:ab,ti,kw OR ‘emotion-focused’:ab,ti,kw OR narrative:ab,ti,kw OR ‘person-centred’:ab,ti,kw OR ‘person-centered’:ab,ti,kw OR ‘Narrative’:ab,ti,kw OR ‘meaning-centered’:ab,ti,kw OR ‘humanistic’:ab,ti,kw OR ‘client-centered’:ab,ti,kw OR ‘meaning-centred’:ab,ti,kw OR ‘client-centred’:ab,ti,kw OR ‘Rogerian’:ab,ti,kw OR ‘Nondirective’:ab,ti,kw OR ‘Non-directive’:ab,ti,kw OR ‘Supportive’:ab,ti,kw OR ‘Life review’:ab,ti,kw OR ‘acceptance and commitment’:ab,ti,kw OR (‘schema’:ab,ti,kw AND brief:ab,ti,kw) OR (‘gestalt’:ab,ti,kw AND brief:ab,ti,kw))) OR ‘behavior therap*’:ab,ti,kw OR ‘behaviors therap*’:ab,ti,kw OR ‘behavioral therap*’:ab,ti,kw OR ‘behaviour therap*’:ab,ti,kw OR ‘behaviours therap*’:ab,ti,kw OR ‘behavioural therap*’:ab,ti,kw OR ‘cognition therap*’:ab,ti,kw OR ‘cognitive therap*’:ab,ti,kw OR ‘behavior treatment*’:ab,ti,kw OR ‘behaviors treatment*’:ab,ti,kw OR ‘behavioral treatment*’:ab,ti,kw OR ‘behaviour treatment*’:ab,ti,kw OR ‘behaviours treatment*’:ab,ti,kw OR ‘behavioural treatment*’:ab,ti,kw OR ‘cognition treatment*’:ab,ti,kw OR ‘cognitive treatment*’:ab,ti,kw OR ‘behavior intervention*’:ab,ti,kw OR ‘behaviors intervention*’:ab,ti,kw OR ‘behavioral intervention*’:ab,ti,kw OR ‘behaviour intervention*’:ab,ti,kw OR ‘behaviours intervention*’:ab,ti,kw OR ‘behavioural intervention*’:ab,ti,kw OR ‘cognition intervention*’:ab,ti,kw OR ‘cognitive intervention*’:ab,ti,kw OR ‘behavior activation*’:ab,ti,kw OR ‘behaviors activation*’:ab,ti,kw OR ‘behavioral activation*’:ab,ti,kw OR ‘behaviour activation*’:ab,ti,kw OR ‘behaviours activation*’:ab,ti,kw OR ‘behavioural activation*’:ab,ti,kw OR exposure:ab,ti,kw

\# Internet-based

'telehealth'/exp OR 'mobile application'/exp OR 'social media'/exp OR 'computer assisted therapy'/de OR 'computer assisted drug therapy'/exp OR 'e-mail'/exp OR 'videoconferencing'/exp OR 'mobile phone'/exp OR 'wearable computer'/exp OR telehealth:ab,ti,kw OR ‘tele health’:ab,ti,kw OR telepsychology:ab,ti,kw OR ‘tele psychology’:ab,ti,kw OR telepsychiatry:ab,ti,kw OR ‘tele psychiatry’:ab,ti,kw OR ‘tele therap*’:ab,ti,kw OR teletherap*:ab,ti,kw OR ‘tele medicine’:ab,ti,kw OR telemedicine:ab,ti,kw OR telecare:ab,ti,kw OR ‘tele care’:ab,ti,kw OR telecommunicat*:ab,ti,kw OR ‘tele communicat*’:ab,ti,kw OR teleconference*:ab,ti,kw OR ‘tele conferenc*’:ab,ti,kw OR videoconferenc*:ab,ti,kw OR ‘video conferenc*’:ab,ti,kw OR computer*:ab,ti,kw OR electronic*:ab,ti,kw OR digital*:ab,ti,kw OR ehealth:ab,ti,kw OR ‘e health’:ab,ti,kw OR ‘e treat*’:ab,ti,kw OR ‘e therap*’:ab,ti,kw OR mhealth:ab,ti,kw OR ‘m health’:ab,ti,kw OR ‘internet based*’:ab,ti,kw OR ‘internet treat*’:ab,ti,kw OR ‘internet intervention*’:ab,ti,kw OR ‘internet counsel*’:ab,ti,kw OR ‘distance counsel*’:ab,ti,kw OR ‘web based*’:ab,ti,kw OR cybercounsel*:ab,ti,kw OR ‘cyber counsel*’:ab,ti,kw OR ‘online treat*’:ab,ti,kw OR ‘online therap*’:ab,ti,kw OR ‘online intervention*’:ab,ti,kw OR ‘online prevention*’:ab,ti,kw OR ‘online counsel*’:ab,ti,kw OR ‘text messag*’:ab,ti,kw OR textmessag*:ab,ti,kw OR SMS:ab,ti,kw OR texting*:ab,ti,kw OR ‘short message service*’:ab,ti,kw OR mobile*:ab,ti,kw OR smartphone*:ab,ti,kw OR ‘cell phone*’:ab,ti,kw OR cellphone*:ab,ti,kw OR ‘cellular phone*’:ab,ti,kw OR blended*:ab,ti,kw OR ‘software app*’:ab,ti,kw OR ‘handheld device*’:ab,ti,kw OR ‘hand held device*’:ab,ti,kw OR iPad*:ab,ti,kw OR iPhone*:ab,ti,kw OR email*:ab,ti,kw OR ‘e-mail*’:ab,ti,kw OR sensor*:ab,ti,kw OR wearable*:ab,ti,kw OR ‘social media*’:ab,ti,kw OR ‘social network*’:ab,ti,kw OR ‘e counsel*’:ab,ti,kw OR ecounsel*:ab,ti,kw OR palmtop*:ab,ti,kw OR telephone*:ab,ti,kw OR WhatsApp:ab,ti,kw OR Twitter:ab,ti,kw OR Facebook:ab,ti,kw OR Instagram:ab,ti,kw OR forum:ab,ti,kw OR chat*:ab,ti,kw OR ‘virtual reality*’:ab,ti,kw OR avatar*:ab,ti,kw OR ‘Conversational agent*’:ab,ti,kw OR ‘virtual coach’:ab,ti,kw OR ‘virtual agent*’:ab,ti,kw OR ‘embodied agent*’:ab,ti,kw OR ‘relational agent*’:ab,ti,kw OR ‘interactive agent*’:ab,ti,kw OR ‘virtual character*’:ab,ti,kw OR ‘virtual human*’:ab,ti,kw OR ‘virtual assistant*’:ab,ti,kw OR VR:ab,ti,kw OR ‘serious game*’:ab,ti,kw OR ‘serious gaming’:ab,ti,kw OR gamification:ab,ti,kw

\# SR + MA filter

'meta analysis'/exp OR 'meta analysis (topic)'/exp OR 'systematic review'/exp OR 'systematic review (topic)'/exp OR metaanaly*:ab,ti,kw OR meta-analy*:ab,ti,kw or metanaly*:ab,ti,kw OR 'cochrane database of systematic reviews'/jt OR prisma:ab,ti,kw OR ‘preferred reporting items’:ab,ti,kw OR prospero:ab,ti,kw OR ‘systematic review’:ab,ti,kw OR ‘umbrella review’:ab,ti,kw OR ‘structured literature review’:ab,ti,kw OR ‘systematic qualitative review’:ab,ti,kw OR ‘systematic quantitative review’:ab,ti,kw OR ‘systematized review’:ab,ti,kw OR ‘systematised review’:ab,ti,kw OR ‘systemic review’:ab,ti,kw OR ‘systematic literature review’:ab,ti,kw OR ‘systematic integrative literature review’:ab,ti,kw OR ‘systematically review’:ab,ti,kw OR ‘scoping literature review’:ab,ti,kw OR ‘scoping review’:ab,ti,kw OR ‘systematic critical review’:ab,ti,kw OR ‘systematic integrative review’:ab,ti,kw OR ‘systematic evidence review’:ab,ti,kw OR ‘systematic integrative literature review’:ab,ti,kw OR ‘systematic mixed studies review’:ab,ti,kw OR ‘systematized literature review’:ab,ti,kw OR ‘systematic overview’:ab,ti,kw OR ‘Systematic narrative review’:ab,ti,kw OR ‘narrative review’:ab,ti,kw OR metasynthes*:ab,ti,kw OR meta-synthes*:ab,ti,kw OR 'clinical trial'/de OR 'double blind procedure'/de OR 'phase 1 clinical trial topic'/de OR 'phase 2 clinical trial topic'/de OR 'phase 3 clinical trial'/de OR 'phase 3 clinical trial topic'/de OR 'randomized controlled trial'/de OR 'single blind procedure'/de OR random:ab,ti,kw OR randomly:ab,ti,kw OR randomised:ab,ti,kw OR randomized:ab,ti,kw OR randomising:ab,ti,kw OR randomizing:ab,ti,kw OR RCT:ab,ti,kw OR ‘clinical trial*’:ab,ti,kw OR ‘controlled trial*’:ab,ti,kw

\# Publicationtype

('article'/it OR 'article in press'/it OR 'conference paper'/it OR 'review'/it OR 'short survey'/it)

\# Timeframe

After 2000

## APA PsycInfo

\# Depression

DE "Major Depression" OR DE "Anaclitic Depression" OR DE "Dysthymic Disorder" OR DE "Endogenous Depression" OR DE "Late Life Depression" OR DE "Postpartum Depression" OR DE "Reactive Depression" OR DE "Recurrent Depression" OR DE "Treatment Resistant Depression" OR DE "Depression (Emotion)" OR TI(“depress*” OR “dysthymi*” OR “mood disorder*” OR “affective disorder*” OR “dysphoric disorder*”) OR AB(“depress*” OR “dysthymi*” OR “mood disorder*” OR “affective disorder*” OR “dysphoric disorder*”) OR KW(“depress*” OR “dysthymi*” OR “mood disorder*” OR “affective disorder*” OR “dysphoric disorder*”)

#Anxiety

DE "Anxiety Disorders" OR DE "Castration Anxiety" OR DE "Generalized Anxiety Disorder" OR DE "Obsessive Compulsive Disorder" OR DE "Panic Attack" OR DE "Panic Disorder" OR DE "Phobias" OR DE "Separation Anxiety Disorder" OR DE "Trichotillomania" OR DE "Phobias" OR DE "Acrophobia" OR DE "Agoraphobia" OR DE "Claustrophobia" OR DE "Ophidiophobia" OR DE "School Phobia" OR DE "Social Phobia" OR DE "Anxiety" OR DE "Anxiety Sensitivity" OR DE "Computer Anxiety" OR DE "Death Anxiety" OR DE "Health Anxiety" OR DE "Mathematics Anxiety" OR DE "Performance Anxiety" OR DE "Social Anxiety" OR DE "Speech Anxiety" OR DE "Test Anxiety" OR DE "Timidity" OR

TI(shyness OR shy OR “anxiet*” OR “agoraphobi*” OR “panic” OR “social phobi*” OR “phobi*” OR “obsessive-compulsive” OR “neurotic disorder*” OR “hoarding*” OR “OCD” OR “neurotic anxiet*” OR “anxious*” OR “emotional disorder*” OR fear OR worry OR worrying OR worries OR GAD OR Arachnophobia OR Ophidiophobia OR Acrophobia OR Cynophobia OR Claustrophobia OR Mysophobia OR Aerophobia OR Trypophobia OR Carcinophobia OR Thanatophobia OR Glossophobia OR Monophobia OR Ornithophobia OR Alektorophobia OR Trypanophobia OR Anthropophobia OR Aquaphobia OR Autophobia OR Hemophobia OR Xenophobia OR Ailurophobia OR Nyctophobia OR Phobophobia OR Philophobia OR Triskaidekaphobia OR Emetophobia OR Entomophobia OR Zoophobia OR Scelerophobia OR Cibophobia OR Tokophobia OR Pseudodysphagia OR Gerascophobia OR Technophobia OR Ergophobia OR Coulrophobia OR Photophobia OR Numerophobia OR Taphophobia) OR AB(shyness OR shy OR “anxiet*” OR “agoraphobi*” OR “panic” OR “social phobi*” OR “phobi*” OR “obsessive-compulsive” OR “neurotic disorder*” OR “hoarding*” OR “OCD” OR “neurotic anxiet*” OR “anxious*” OR “emotional disorder*” OR fear OR worry OR worrying OR worries OR GAD OR Arachnophobia OR Ophidiophobia OR Acrophobia OR Cynophobia OR Claustrophobia OR Mysophobia OR Aerophobia OR Trypophobia OR Carcinophobia OR Thanatophobia OR Glossophobia OR Monophobia OR Ornithophobia OR Alektorophobia OR Trypanophobia OR Anthropophobia OR Aquaphobia OR Autophobia OR Hemophobia OR Xenophobia OR Ailurophobia OR Nyctophobia OR Phobophobia OR Philophobia OR Triskaidekaphobia OR Emetophobia OR Entomophobia OR Zoophobia OR Scelerophobia OR Cibophobia OR Tokophobia OR Pseudodysphagia OR Gerascophobia OR Technophobia OR Ergophobia OR Coulrophobia OR Photophobia OR Numerophobia OR Taphophobia) OR KW(shyness OR shy OR “anxiet*” OR “agoraphobi*” OR “panic” OR “social phobi*” OR “phobi*” OR “obsessive-compulsive” OR “neurotic disorder*” OR “hoarding*” OR “OCD” OR “neurotic anxiet*” OR “anxious*” OR “emotional disorder*” OR fear OR worry OR worrying OR worries OR GAD OR Arachnophobia OR Ophidiophobia OR Acrophobia OR Cynophobia OR Claustrophobia OR Mysophobia OR Aerophobia OR Trypophobia OR Carcinophobia OR Thanatophobia OR Glossophobia OR Monophobia OR Ornithophobia OR Alektorophobia OR Trypanophobia OR Anthropophobia OR Aquaphobia OR Autophobia OR Hemophobia OR Xenophobia OR Ailurophobia OR Nyctophobia OR Phobophobia OR Philophobia OR Triskaidekaphobia OR Emetophobia OR Entomophobia OR Zoophobia OR Scelerophobia OR Cibophobia OR Tokophobia OR Pseudodysphagia OR Gerascophobia OR Technophobia OR Ergophobia OR Coulrophobia OR Photophobia OR Numerophobia OR Taphophobia)

\# Psychotherapy

DE "Psychotherapy" OR DE "Adlerian Psychotherapy" OR DE "Adolescent Psychotherapy" OR DE "Affirmative Therapy" OR DE "Analytical Psychotherapy" OR DE "Autogenic Training" OR DE "Brief Psychotherapy" OR DE "Brief Relational Therapy" OR DE "Child Psychotherapy" OR DE "Client Centered Therapy" OR DE "Couples Therapy" OR DE "Eclectic Psychotherapy" OR DE "Emotion Focused Therapy" OR DE "Existential Therapy" OR DE "Experiential Psychotherapy" OR DE "Expressive Psychotherapy" OR DE "Eye Movement Desensitization Therapy" OR DE "Feminist Therapy" OR DE "Gestalt Therapy" OR DE "Empty Chair Technique" OR DE "Group Psychotherapy" OR DE "Encounter Group Therapy" OR DE "Therapeutic Community" OR DE "Guided Imagery" OR DE "Humanistic Psychotherapy" OR DE "Client Centered Therapy" OR DE "Individual Psychotherapy" OR DE "Insight Therapy" OR DE "Integrative Psychotherapy" OR DE "Schema Therapy" OR DE "Interpersonal Psychotherapy" OR DE "Logotherapy" OR DE "Narrative Therapy" OR DE "Network Therapy" OR DE "Persuasion Therapy" OR DE "Primal Therapy" OR DE "Psychodrama" OR DE "Psychodynamic Psychotherapy" OR DE "Psychotherapeutic Counseling" OR DE "Family Therapy" OR DE "Psychotherapeutic Techniques" OR DE "Active Listening" OR DE "Animal Assisted Therapy" OR DE "Autogenic Training" OR DE "Brief Relational Therapy" OR DE "Centering" OR DE "Cotherapy" OR DE "Dream Analysis" OR DE "Empty Chair Technique" OR DE "Ericksonian Psychotherapy" OR DE "Free Association" OR DE "Guided Imagery" OR DE "Life Review" OR DE "Mirroring" OR DE "Morita Therapy" OR DE "Motivational Interviewing" OR DE "Mutual Storytelling Technique" OR DE "Network Therapy" OR DE "Paradoxical Techniques" OR DE "Psychodrama" OR DE "Rational Emotive Behavior Therapy" OR DE "Reality Therapy" OR DE "Relationship Therapy" OR DE "Solution Focused Therapy" OR DE "Strategic Therapy" OR DE "Strategic Family Therapy" OR DE "Supportive Psychotherapy" OR DE "Transactional Analysis" OR DE "Cognitive Therapy" OR TI(psychotherap* OR cbt OR counselling OR counseling OR “Eye Movement Desensitization Reprocessing” OR “Eye Movement Desensitization and Reprocessing” OR “Eye Movement Desensitisation Reprocessing” OR “Eye Movement Desensitisation and Reprocessing” OR EMDR OR “Bibliotherap*” OR mindfulness OR “Autogenic Training” OR Logotherap* OR "cognitive restructuring" OR "self-control training*" OR "assertiveness training" OR ((therap* OR treatment* OR intervention*) AND (“brief psychodynamic” OR “short psychodynamic” OR "problem-solving" OR "compassion-focused" OR "compassion-focussed" OR “compassion-based” OR constructivist* OR metacognitive OR “meta-cognitive” OR "solution-focused" OR "solution-focussed" OR "self-control” OR psychosocial OR “peer support“ OR “task-shifted” OR Relaxation OR “dialectical behavior” OR “emotion-focused” OR narrative OR “person-centred” OR “person-centered” OR “Narrative” OR “meaning-centered” OR “humanistic” OR “client-centered” OR “meaning-centred” OR “client-centred” OR “Rogerian” OR “Nondirective” OR “Non-directive” OR “Supportive” OR “Life review” OR "acceptance and commitment" OR (“schema” AND brief) OR (“gestalt” AND brief))) OR "behavior therap*" OR "behaviors therap*" OR "behavioral therap*" OR "behaviour therap*" OR "behaviours therap*" OR "behavioural therap*" OR "cognition therap*" OR “cognitive therap*” OR "behavior treatment*" OR "behaviors treatment*" OR "behavioral treatment*" OR "behaviour treatment*" OR "behaviours treatment*" OR "behavioural treatment*" OR "cognition treatment*" OR “cognitive treatment*” OR "behavior intervention*" OR "behaviors intervention*" OR "behavioral intervention*" OR "behaviour intervention*" OR "behaviours intervention*" OR "behavioural intervention*" OR "cognition intervention*" OR “cognitive intervention*” OR "behavior activation*" OR "behaviors activation*" OR "behavioral activation*" OR "behaviour activation*" OR "behaviours activation*" OR "behavioural activation*" OR exposure) OR AB((psychotherap* OR cbt OR counselling OR counseling OR “Eye Movement Desensitization Reprocessing” OR “Eye Movement Desensitization and Reprocessing” OR “Eye Movement Desensitisation Reprocessing” OR “Eye Movement Desensitisation and Reprocessing” OR EMDR OR “Bibliotherap*” OR mindfulness OR “Autogenic Training” OR Logotherap* OR "cognitive restructuring" OR "self-control training*" OR "assertiveness training" OR ((therap* OR treatment* OR intervention*) AND (“brief psychodynamic” OR “short psychodynamic” OR "problem-solving" OR "compassion-focused" OR "compassion-focussed" OR “compassion-based” OR constructivist* OR metacognitive OR “meta-cognitive” OR "solution-focused" OR "solution-focussed" OR "self-control” OR psychosocial OR “peer support“ OR “task-shifted” OR Relaxation OR “dialectical behavior” OR “emotion-focused” OR narrative OR “person-centred” OR “person-centered” OR “Narrative” OR “meaning-centered” OR “humanistic” OR “client-centered” OR “meaning-centred” OR “client-centred” OR “Rogerian” OR “Nondirective” OR “Non-directive” OR “Supportive” OR “Life review” OR "acceptance and commitment" OR (“schema” AND brief) OR (“gestalt” AND brief))) OR "behavior therap*" OR "behaviors therap*" OR "behavioral therap*" OR "behaviour therap*" OR "behaviours therap*" OR "behavioural therap*" OR "cognition therap*" OR “cognitive therap*” OR "behavior treatment*" OR "behaviors treatment*" OR "behavioral treatment*" OR "behaviour treatment*" OR "behaviours treatment*" OR "behavioural treatment*" OR "cognition treatment*" OR “cognitive treatment*” OR "behavior intervention*" OR "behaviors intervention*" OR "behavioral intervention*" OR "behaviour intervention*" OR "behaviours intervention*" OR "behavioural intervention*" OR "cognition intervention*" OR “cognitive intervention*” OR "behavior activation*" OR "behaviors activation*" OR "behavioral activation*" OR "behaviour activation*" OR "behaviours activation*" OR "behavioural activation*" OR exposure) OR KW(psychotherap* OR cbt OR counselling OR counseling OR “Eye Movement Desensitization Reprocessing” OR “Eye Movement Desensitization and Reprocessing” OR “Eye Movement Desensitisation Reprocessing” OR “Eye Movement Desensitisation and Reprocessing” OR EMDR OR “Bibliotherap*” OR mindfulness OR “Autogenic Training” OR Logotherap* OR "cognitive restructuring" OR "self-control training*" OR "assertiveness training" OR ((therap* OR treatment* OR intervention*) AND (“brief psychodynamic” OR “short psychodynamic” OR "problem-solving" OR "compassion-focused" OR "compassion-focussed" OR “compassion-based” OR constructivist* OR metacognitive OR “meta-cognitive” OR "solution-focused" OR "solution-focussed" OR "self-control” OR psychosocial OR “peer support“ OR “task-shifted” OR Relaxation OR “dialectical behavior” OR “emotion-focused” OR narrative OR “person-centred” OR “person-centered” OR “Narrative” OR “meaning-centered” OR “humanistic” OR “client-centered” OR “meaning-centred” OR “client-centred” OR “Rogerian” OR “Nondirective” OR “Non-directive” OR “Supportive” OR “Life review” OR "acceptance and commitment" OR (“schema” AND brief) OR (“gestalt” AND brief))) OR "behavior therap*" OR "behaviors therap*" OR "behavioral therap*" OR "behaviour therap*" OR "behaviours therap*" OR "behavioural therap*" OR "cognition therap*" OR “cognitive therap*” OR "behavior treatment*" OR "behaviors treatment*" OR "behavioral treatment*" OR "behaviour treatment*" OR "behaviours treatment*" OR "behavioural treatment*" OR "cognition treatment*" OR “cognitive treatment*” OR "behavior intervention*" OR "behaviors intervention*" OR "behavioral intervention*" OR "behaviour intervention*" OR "behaviours intervention*" OR "behavioural intervention*" OR "cognition intervention*" OR “cognitive intervention*” OR "behavior activation*" OR "behaviors activation*" OR "behavioral activation*" OR "behaviour activation*" OR "behaviours activation*" OR "behavioural activation*" OR exposure)

\# Internet-based

DE "Computer Assisted Therapy" OR DE "Online Therapy" OR DE "Teleconsultation" OR DE "Telepsychiatry" OR DE "Telepsychology" OR DE "Telerehabilitation" OR DE "Digital Interventions" OR DE "Telemedicine" OR DE "Videoconferencing" OR DE "Telecommunications Media" OR DE "Teleconferencing" OR DE "Smartphones" OR DE "Mobile Applications" OR DE "Mobile Devices" OR DE "Mobile Phones" OR DE "Tablet Computers" OR DE "Mobile Health" OR DE "Text Messaging" OR DE "Wearable Devices" OR DE "Virtual Reality" OR DE "Augmented Reality" OR TI(telehealth OR “tele-health” OR telepsychology OR “tele-psychology” OR telepsychiatry OR “tele-psychiatry” OR “tele-therap*” OR teletherap* OR “tele-medicine” OR telemedicine OR telecare OR “tele-care” OR telecommunicat* OR “tele-communicat*” OR teleconference* OR “tele-conferenc*” OR videoconferenc* OR “video-conferenc*” OR computer* OR electronic* OR digital* OR ehealth OR “e-health” OR “e-treat*” OR “e-therap*” OR mhealth OR “m-health” OR “internet-based*” OR “internet treat*” OR “internet intervention*” OR “internet counsel*” OR “distance counsel*” OR “web-based*” OR cybercounsel* OR “cyber-counsel*” OR “online treat*” OR “online therap*” OR “online intervention*” OR “online prevention*” OR “online counsel*” OR “text-messag*” OR textmessag* OR SMS OR texting* OR “short message service*” OR mobile* OR smartphone* OR “cell-phone*” OR cellphone* OR “cellular phone*” OR blended* OR “software app*” OR “handheld device*” OR “hand held device*” OR iPad* OR iPhone* OR email* OR “e-mail*” OR sensor* OR wearable* OR “social media*” OR “social network*” OR “e-counsel*” OR ecounsel* OR palmtop* OR telephone* OR WhatsApp OR Twitter OR Facebook OR Instagram OR forum OR chat* OR “virtual reality*” OR avatar* OR “Conversational agent*” OR “virtual coach” OR “virtual agent*” OR “embodied agent*” OR “relational agent*” OR “interactive agent*” OR “virtual character*” OR “virtual human*” OR “virtual assistant*” OR VR OR “serious game*” OR “serious gaming” OR gamification) OR AB(telehealth OR “tele-health” OR telepsychology OR “tele-psychology” OR telepsychiatry OR “tele-psychiatry” OR “tele-therap*” OR teletherap* OR “tele-medicine” OR telemedicine OR telecare OR “tele-care” OR telecommunicat* OR “tele-communicat*” OR teleconference* OR “tele-conferenc*” OR videoconferenc* OR “video-conferenc*” OR computer* OR electronic* OR digital* OR ehealth OR “e-health” OR “e-treat*” OR “e-therap*” OR mhealth OR “m-health” OR “internet-based*” OR “internet treat*” OR “internet intervention*” OR “internet counsel*” OR “distance counsel*” OR “web-based*” OR cybercounsel* OR “cyber-counsel*” OR “online treat*” OR “online therap*” OR “online intervention*” OR “online prevention*” OR “online counsel*” OR “text-messag*” OR textmessag* OR SMS OR texting* OR “short message service*” OR mobile* OR smartphone* OR “cell-phone*” OR cellphone* OR “cellular phone*” OR blended* OR “software app*” OR “handheld device*” OR “hand held device*” OR iPad* OR iPhone* OR email* OR “e-mail*” OR sensor* OR wearable* OR “social media*” OR “social network*” OR “e-counsel*” OR ecounsel* OR palmtop* OR telephone* OR WhatsApp OR Twitter OR Facebook OR Instagram OR forum OR chat* OR “virtual reality*” OR avatar* OR “Conversational agent*” OR “virtual coach” OR “virtual agent*” OR “embodied agent*” OR “relational agent*” OR “interactive agent*” OR “virtual character*” OR “virtual human*” OR “virtual assistant*” OR VR OR “serious game*” OR “serious gaming” OR gamification) OR KW(telehealth OR “tele-health” OR telepsychology OR “tele-psychology” OR telepsychiatry OR “tele-psychiatry” OR “tele-therap*” OR teletherap* OR “tele-medicine” OR telemedicine OR telecare OR “tele-care” OR telecommunicat* OR “tele-communicat*” OR teleconference* OR “tele-conferenc*” OR videoconferenc* OR “video-conferenc*” OR computer* OR electronic* OR digital* OR ehealth OR “e-health” OR “e-treat*” OR “e-therap*” OR mhealth OR “m-health” OR “internet-based*” OR “internet treat*” OR “internet intervention*” OR “internet counsel*” OR “distance counsel*” OR “web-based*” OR cybercounsel* OR “cyber-counsel*” OR “online treat*” OR “online therap*” OR “online intervention*” OR “online prevention*” OR “online counsel*” OR “text-messag*” OR textmessag* OR SMS OR texting* OR “short message service*” OR mobile* OR smartphone* OR “cell-phone*” OR cellphone* OR “cellular phone*” OR blended* OR “software app*” OR “handheld device*” OR “hand held device*” OR iPad* OR iPhone* OR email* OR “e-mail*” OR sensor* OR wearable* OR “social media*” OR “social network*” OR “e-counsel*” OR ecounsel* OR palmtop* OR telephone* OR WhatsApp OR Twitter OR Facebook OR Instagram OR forum OR chat* OR “virtual reality*” OR avatar* OR “Conversational agent*” OR “virtual coach” OR “virtual agent*” OR “embodied agent*” OR “relational agent*” OR “interactive agent*” OR “virtual character*” OR “virtual human*” OR “virtual assistant*” OR VR OR “serious game*” OR “serious gaming” OR gamification)

\# SR + MA filter

TI(metaanaly* OR meta-analy* or metanaly* OR prisma OR “preferred reporting items” OR prospero OR “systematic review” OR “umbrella review” OR “structured literature review” OR “systematic qualitative review” OR “systematic quantitative review” OR “systematic search and review” OR “systematized review” OR “systematised review” OR “systemic review” OR “systematic literature review” OR “systematic integrative literature review” OR “systematically review” OR “scoping literature review” OR “scoping review” OR “systematic critical review” OR “systematic integrative review” OR “systematic evidence review” OR “systematic integrative literature review” OR “systematic mixed studies review” OR “systematized literature review” OR “systematic overview” OR “Systematic narrative review” OR “narrative review” OR metasynthes* OR meta-synthes* OR random OR randomly OR randomised OR randomized OR randomising OR randomizing OR RCT OR “clinical trial*” OR “controlled trial*”) OR AB(metaanaly* OR meta-analy* or metanaly* OR prisma OR “preferred reporting items” OR prospero OR “systematic review” OR “umbrella review” OR “structured literature review” OR “systematic qualitative review” OR “systematic quantitative review” OR “systematic search and review” OR “systematized review” OR “systematised review” OR “systemic review” OR “systematic literature review” OR “systematic integrative literature review” OR “systematically review” OR “scoping literature review” OR “scoping review” OR “systematic critical review” OR “systematic integrative review” OR “systematic evidence review” OR “systematic integrative literature review” OR “systematic mixed studies review” OR “systematized literature review” OR “systematic overview” OR “Systematic narrative review” OR “narrative review” OR metasynthes* OR meta-synthes* OR random OR randomly OR randomised OR randomized OR randomising OR randomizing OR RCT OR “clinical trial*” OR “controlled trial*”) OR KW(metaanaly* OR meta-analy* or metanaly* OR prisma OR “preferred reporting items” OR prospero OR “systematic review” OR “umbrella review” OR “structured literature review” OR “systematic qualitative review” OR “systematic quantitative review” OR “systematic search and review” OR “systematized review” OR “systematised review” OR “systemic review” OR “systematic literature review” OR “systematic integrative literature review” OR “systematically review” OR “scoping literature review” OR “scoping review” OR “systematic critical review” OR “systematic integrative review” OR “systematic evidence review” OR “systematic integrative literature review” OR “systematic mixed studies review” OR “systematized literature review” OR “systematic overview” OR “Systematic narrative review” OR “narrative review” OR metasynthes* OR random OR randomly OR randomised OR randomized OR randomising OR randomizing OR RCT OR “clinical trial*” OR “controlled trial*”)

\# Publication type

Academic journals

\# Timeframe

After 2000

# **Appendix D. Study-level Variables Extracted from Published Studies**

We collected:

- **Study information:** Year of publication, country, income of country, outcomes assessed.
- **Participant characteristics:** Number of participants, percentage of female participants, mean age, age group, type of recruitment, manner of diagnosis.
- **Condition information:** Type of intervention, type of control, name of intervention, type and amount of support, existence of a forum available for this intervention, number of sessions planned and mean.
- **Data to calculate effect sizes:** The mean (*M*) score in depressive symptoms in each of the compared groups, their standard deviations (*SD*), and number of participants. We used other statistics, such as standard errors (*SEs*) or 95% confidence intervals (95% CIs) to replace non-reported *SD*s, and change scores, dichotomous, or other statistic data (such as *F*-statistic) were collected if continuous data (i.e., *Ms* and *SDs*) were not reported. Where possible, intention-to-treat (ITT) data were extracted over completers-only.

# **Appendix E. Requested Individual Participant Data Variables**

If available, we asked the contributing authors to provide us with the following variables:

- **Socio-demographic characteristics:** Gender, age, ethnicity, education level, relationship status, employment status.
- **Clinical characteristics:** Depressive symptoms at baseline, mid-treatment, post-treatment, and follow-up, anxiety symptoms, quality of life, distress, previous treatment, comorbid mental disorders, use of psychoactive medication, substance use, insomnia, specific phobias, childhood adversities.
- **Information about pregnancy and the infant:** Type of delivery, gestational age at birth, pregnancy complications, satisfaction with breastfeeding, birth weight, birth trauma, infant physical and mental health.
- **Intervention information:** Details about intervention or control, time spent in the intervention, time spent with the therapist, number of messages exchanged with the therapist, number of calls with the therapist, intervention duration for each participant, number of internet-based modules completed, number of video and face-to-face sessions completed, expectations about the intervention, satisfaction with the intervetion, system usability, intervention fidelity, treatment burden.
- **Information about the mother’s partner:** Any indicators of mental or physical health.
- **Psycho-social outcomes and relationships:** Social support, bonding with the infant, presence of domestic violence, work and social adjustment, global and social functioning.

# **Appendix F. Conventional Meta-analysis – Analysis Details**

Hedges’ *g* was calculated to indicate the standardized mean difference in depressive symptoms between the intervention and the control at post-treatment, and to adjust for small samples by subtracting the mean (*M*) total score of the intervention arm from the mean total score of the control arm and dividing the results by the standard deviation (*SD*) pooled from *SDs* of both arms (Hedges & Olkin, 2014). The effect size pooled from all included studies, using all reported outcome measures per study, was then calculated using the random effects model, as statistical heterogeneity among studies was expected. For aggregate effects within comparisons, the assumption of an intra-study correlation coefficient of *ρ* = .5 was applied. Between-study heterogeneity variance was estimated using restricted maximum likelihood, CIs were calculated and significance tests of the overall effect were performed using the robust variance estimation (RVE) and the Knapp-Hartung method (IntHout et al., 2014). *I^2^*-statistics and its 95%CI expressed heterogeneity, where no, low, moderate and high heterogeneity are represented as 0, 25, 50, and 75% (Higgins et al., 2003). We further calculated the 95% prediction intervals (95% PIs), indicating the interval in which we would expect the true effect size in 95% of future observations (Borenstein et al., 2021; Borenstein et al., 2017).

Sensitivity analyses included (1) using only the largest, (2) and the smallest effect size per study in case of multiple comparisons, (3) including only studies with low risk of bias, and (4) a three-level correlated and hierarchical effects (CHE) model (Pustejovsky & Tipton, 2022), where an intra-study correlation of *ρ* = .5 was assumed, parameter tests and CIs were calculated by RVE, and heterogeneity was calculated with a multilevel extension of *I^2^* (Cheung, 2014; Viechtbauer & Cheung, 2010). We also conducted analyses comparing the effects of interventions in subgroups of studies, namely: (1) studies which did versus did not provide IPD, (2) studies testing CBT versus other interventions, (3) studies using care-as-usual (CAU) versus waitlist condition (WL), as only two studies used other types of control, (4) studies testing guided versus self-guided interventions, (5) studies recruiting participants based on a diagnosis of a mood disorder derived from a diagnostic interview versus studies using a cut-off on a self-report measure, and (6) studies conducted in Western (i.e., Europe and European-settled) versus non-Western countries (Hanson, 2007). We also conducted a multivariate meta-regression analysis, where we assessed the relationship between the effect size and whether we succeeded in collecting the IPD, while controlling for risk of bias, type of control, and region of conduct (conduct in a Western [i.e., Europe or European-settled] country) (Hanson, 2007).

# **Appendix G. Multiple Imputation**

## Details of the Analysis

First, missingness (Harrer et al., 2023), influx and outflux patterns (Van Buuren, 2012), and association between variables and missingness (Carpenter & Smuk, 2021) were explored, to understand which variables should be included in the MICE procedure. For this decision, all auxiliary variables of interest (i.e., investigated effect modifiers) as well as any additional assessments of the outcome measure (such as mid-treatment and follow-up assessments) were considered. MICE then used predictive mean matching to estimate missing values (Morris et al., 2014). The imputations were then checked for potential problems using trace and density plots (Harrer et al., 2023).

## Visualization of Imputation Matrices per Study

In imputation matrices, variables shown in the rows (i.e., on the y-axis) are imputed using information from variables lying in the columns (i.e., on the x-axis). Using this visualization, imputation matrix thus shows for every variable with missing values which predictor information was used to handle missing data, namely the variables whose cells overlapping with the row of interest are in blue.

1)*Danaher, 2022*
2)*Fonseca, 2020*
3)*Forsell, 2017*
4)*Franco, 2024*
5)*Milgrom, 2016*
6)*Milgrom, 2021*
7)*Pugh, 2016*
8)*Sun, 2021*
9)*Zuccolo, 2024*

# **Appendix H. IPDMA – Analysis Detail and Sensitivity Analyses**

To calculate the Reliable Change Index, we used the original, i.e., non-converted, scales, a baseline standard deviation for each study, and a test-retest reliability coefficient of the outcome measure, namely 0.75 (Wang et al., 2021) for the Patient Health Questionnaire-9 (PHQ-9) (Kroenke et al., 2001), and 0.92 (Kernot et al., 2015) for the Edinburgh Postnatal Depression Scale (EPDS) (Cox & Holden, 2003).

Sensitivity analyses for the effectiveness analysis were conducted (1) including only observed data (i.e., complete case analysis), (2) using one-stage approach, where the IPD from all included studies were merged, participants were nested within studies, and multilevel-mixed effect regression were used, with random intercepts model with a random effect for each trial and fixed effect for each condition (Riley et al., 2023), (3) using response rates (50% reduction of baseline depression symptoms) and (4) remission (scoring below a cut-off indicating mild depressive symptoms) as outcomes, the latter two of which were calculated using the scales originally used in each trial. Response and remission analyses were conducted using logistic regression (Williamson et al., 2013) with regression standardization to provide relative risks (*RR*s) (Morris et al., 2022; Naimi et al., 2017; Robins, 1986; Vansteelandt & Keiding, 2011), in order to avoid artificial inflation of the effect size (Groenwold et al., 2011; Jiang et al., 2017). For deterioration, a sensitivity analysis was conducted with any deterioration, meaning an increase of one or more points on a depressive symptom scale from baseline to post-treatment (Karyotaki et al., 2018). Adherence analysis was re-run using Poisson regression, where the number of modules offered was chosen as an offset (Knudsen, 1992), and separately for studies focused on guided interventions, as adherence and its prognostic factors may differ in them from self-guided interventions (Tong et al., 2026). Unfortunately, the insufficient number of studies with self-guided format prevented us from running the same analysis in this subgroup.

The reliable deterioration analysis was originally supposed to be run using logistic regression with regression standardization, similarly to other dichotomous outcomes. However, deterioration was such as rare outcome that these models failed to converge. Similarly, the analysis was not possible using Firth’s logistic regression for rare events (Firth, 1993), therefore, we decided to analyze the data with Bayesian regression, as described in the main body of the study.

# **Appendix I. PRISMA IPD Flow Diagram**

Records identified through database screening:

(n= 25,989)

Databases: PubMed (n = 9,438); Embase (n = 11,935); PsycINFO (n = 4,616)

Records identified from:

Existing Depression database (n= 8)

Existing Anxiety database (n=4)

Known references (n=3)

Non RCTs MA [Etzelmueller, 2020] (n = 6)

**Available data**

**Obtaining data**

**Identification**

**Screening**

Records for which IPD were provided (n=10)

Data provided for all included participants (n=1128)

Eligible records (n=18)

Records for which IPD were sought (n=18)

Records assessed for eligibility on a full-text basis (n=1,798)

Records screened after duplicates removed (n=14,847)

Records excluded at title/abstract screening (n=13,070)

Records for which aggregate data were available from the published manuscripts (n=8)

Number of participants (n=1,155)

Reports excluded (n=1780):

RCTs not targeting perinatal depression (n=178)

Not found (n=14)

Companion papers (n=195)

Depression was not assessed as an eligibility criterion in primary studies (n=520)

Not internet-based (n=142)

Effect size not reported/cannot be calculated (n=44)

Other (n=49)

No psychotherapy for depression (n=37)

No control group of interest (n=69)

Observational studies (n=87)

Protocol papers (n= 300)

Systematic reviews and meta-analyses (n=145)

**Eligibility**

**Analysed data**

Records for which IPD were not provided (n=8)

Number of participants of non-available studies (n=1,984)

Reasons for exclusion:

Authors could not be reached (n=4)

Loss of access or loss of important data (n=2)

Data-sharing agreements not completed in time (n=2)

**IPD for main analysis**

Number of studies included in analysis (n=9)

Number of participants included in analysis (n=1094)

Number of studies excluded from the analysis (n=1)

Number participants excluded (n=34)

**Aggregate data for conventional MA**

Records for which aggregate data were available (n=17)

Number of participants (n=2,136)

Number participants excluded (n=113)

# **Appendix J. References of the Studies Included in the Conventional Meta-Analysis**

Dai, J., Huang, Y., Liu, J., Sun, Y., Fan, X., Tu, Y., ... & Liu, Y. (2025). Effect of the thinking	healthy programme-based internet intervention model for maternal perinatal	depression: A pilot study. *Journal of Affective Disorders*, *374*, 294-302.

Danaher, B. G., Seeley, J. R., Silver, R. K., Tyler, M. S., Kim, J. J., La Porte, L. M., ... & Gau,	J. M. (2023). Trial of a patient-directed eHealth program to ameliorate perinatal	depression: the MomMoodBooster2 practical effectiveness study. *American journal of	obstetrics and gynecology*, *228*(4), 453-e1.

Fonseca, A., Alves, S., Monteiro, F., Gorayeb, R., & Canavarro, M. C. (2020). Be a mom, a	web-based intervention to prevent postpartum depression: results from a pilot	randomized controlled trial. *Behavior Therapy*, *51*(4), 616-633.

Forsell, E., Bendix, M., Holländare, F., von Schultz, B. S., Nasiell, J., Blomdahl-Wetterholm,	M., ... & Kaldo, V. (2017). Internet delivered cognitive behavior therapy for antenatal	depression: A randomised controlled trial. *Journal of affective disorders*, *221*, 56-64.

Franco, P., Olhaberry, M., Kelders, S., Muzard, A., & Cuijpers, P. (2024). Guided web app	intervention for reducing symptoms of depression in postpartum women: Results of a	feasibility randomized controlled trial. *Internet interventions*, *36*, 100744.

Hassdenteufel, K., Müller, M., Abele, H., Brucker, S. Y., Graf, J., Zipfel, S., ... & Wallwiener,	S. (2023). Using an Electronic Mindfulness-based Intervention (eMBI) to improve	maternal mental health during pregnancy: Results from a randomized controlled	trial. *Psychiatry Research*, *330*, 115599.

Jannati, N., Mazhari, S., Ahmadian, L., & Mirzaee, M. (2020). Effectiveness of an app-based	cognitive behavioral therapy program for postpartum depression in primary care: A	randomized controlled trial. *International Journal of Medical Informatics*, *141*,	104145.

Milgrom, J., Danaher, B. G., Gemmill, A. W., Holt, C., Holt, C. J., Seeley, J. R., ... & Ericksen,	J. (2016). Internet cognitive behavioral therapy for women with postnatal depression: a	randomized controlled trial of MumMoodBooster. *Journal of medical Internet	research*, *18*(3), e4993.

Milgrom, J., Danaher, B. G., Seeley, J. R., Holt, C. J., Holt, C., Ericksen, J., ... & Gemmill, A.	W. (2021). Internet and face-to-face cognitive behavioral therapy for postnatal	depression compared with treatment as usual: randomized controlled trial of	MumMoodBooster. *Journal of medical Internet research*, *23*(12), e17185.

O'Mahen, H. A., Richards, D. A., Woodford, J., Wilkinson, E., McGinley, J., Taylor, R. S., &	Warren, F. C. (2014). Netmums: a phase II randomized controlled trial of a guided	Internet behavioural activation treatment for postpartum depression. *Psychological	medicine*, *44*(8), 1675-1689.

O'Mahen, H. A., Woodford, J., McGinley, J., Warren, F. C., Richards, D. A., Lynch, T. R., &	Taylor, R. S. (2013). Internet-based behavioral activation—Treatment for postnatal	depression (Netmums): A randomized controlled trial. *Journal of affective	disorders*, *150*(3), 814-822.

Pugh, N. E., Hadjistavropoulos, H. D., & Dirkse, D. (2016). A randomised controlled trial of	therapist-assisted, internet-delivered cognitive behavior therapy for women with	maternal depression. *PloS one*, *11*(3), e0149186.

Sawyer, A., Kaim, A., Le, H. N., McDonald, D., Mittinty, M., Lynch, J., & Sawyer, M. (2019).	The effectiveness of an app-based nurse-moderated program for new mothers with	depression and parenting problems (eMums Plus): pragmatic randomized controlled	trial. *Journal of medical Internet research*, *21*(6), e13689.

Seo, J. M., Kim, S. J., Na, H., Kim, J. H., & Lee, H. (2022, October). Effectiveness of a mobile	application for postpartum depression self-management: evidence from a randomised	controlled trial in South Korea. In *Healthcare* (Vol. 10, No. 11, p. 2185). MDPI.

Sun, Y., Li, Y., Wang, J., Chen, Q., Bazzano, A. N., & Cao, F. (2021). Effectiveness of	smartphone-based mindfulness training on maternal perinatal depression: randomized	controlled trial. *Journal of Medical Internet Research*, *23*(1), e23410.

Vigod, S. N., Slyfield Cook, G., Macdonald, K., Hussain‐Shamsy, N., Brown, H. K., de	Oliveira, C., ... & Dennis, C. L. (2021). Mother Matters: Pilot randomized wait‐list	controlled trial of an online therapist‐facilitated discussion board and support group for	postpartum depression symptoms. *Depression and anxiety*, *38*(8), 816-825.

Zuccolo, P. F., Brunoni, A. R., Borja, T., Matijasevich, A., Polanczyk, G. V., & Fatori, D.	(2024). Efficacy of a standalone smartphone application to treat postnatal depression:	a randomized controlled trial. *Psychotherapy and Psychosomatics*, *93*(6), 412-424.

# **Appendix K. References of the Studies Included in the Individual Participant Data Meta-Analysis**

Danaher, B. G., Seeley, J. R., Silver, R. K., Tyler, M. S., Kim, J. J., La Porte, L. M., ... & Gau,	J. M. (2023). Trial of a patient-directed eHealth program to ameliorate perinatal	depression: the MomMoodBooster2 practical effectiveness study. *American journal of	obstetrics and gynecology*, *228*(4), 453-e1.

Fonseca, A., Alves, S., Monteiro, F., Gorayeb, R., & Canavarro, M. C. (2020). Be a mom, a	web-based intervention to prevent postpartum depression: results from a pilot	randomized controlled trial. *Behavior Therapy*, *51*(4), 616-633.

Forsell, E., Bendix, M., Holländare, F., von Schultz, B. S., Nasiell, J., Blomdahl-Wetterholm,	M., ... & Kaldo, V. (2017). Internet delivered cognitive behavior therapy for antenatal	depression: A randomised controlled trial. *Journal of affective disorders*, *221*, 56-64.

Franco, P., Olhaberry, M., Kelders, S., Muzard, A., & Cuijpers, P. (2024). Guided web app	intervention for reducing symptoms of depression in postpartum women: Results of a	feasibility randomized controlled trial. *Internet interventions*, *36*, 100744.

Milgrom, J., Danaher, B. G., Gemmill, A. W., Holt, C., Holt, C. J., Seeley, J. R., ... & Ericksen,	J. (2016). Internet cognitive behavioral therapy for women with postnatal depression: a	randomized controlled trial of MumMoodBooster. *Journal of medical Internet	research*, *18*(3), e4993.

Milgrom, J., Danaher, B. G., Seeley, J. R., Holt, C. J., Holt, C., Ericksen, J., ... & Gemmill, A.	W. (2021). Internet and face-to-face cognitive behavioral therapy for postnatal	depression compared with treatment as usual: randomized controlled trial of	MumMoodBooster. *Journal of medical Internet research*, *23*(12), e17185.

Pugh, N. E., Hadjistavropoulos, H. D., & Dirkse, D. (2016). A randomised controlled trial of	therapist-assisted, internet-delivered cognitive behavior therapy for women with	maternal depression. *PloS one*, *11*(3), e0149186.

Sun, Y., Li, Y., Wang, J., Chen, Q., Bazzano, A. N., & Cao, F. (2021). Effectiveness of	smartphone-based mindfulness training on maternal perinatal depression: randomized	controlled trial. *Journal of Medical Internet Research*, *23*(1), e23410.

Zuccolo, P. F., Brunoni, A. R., Borja, T., Matijasevich, A., Polanczyk, G. V., & Fatori, D.	(2024). Efficacy of a standalone smartphone application to treat postnatal depression:	a randomized controlled trial. *Psychotherapy and Psychosomatics*, *93*(6), 412-424.

# **Appendix L. Risk of Bias of Studies Included in the IPDMA**

## Risk of bias traffic light plot

## Risk of bias summary plot

# **Appendix M. Risk of Bias of Studies Included in the Conventional Meta-analysis**

## Risk of bias traffic light plot

## Risk of bias summary plot

# **Appendix N. Results of Conventional Meta-analysis**

|  | *k* | *g* | *95% CI* | *I^2^* | *95% CI* | *95% PI* | *p* | *NNT* |
| --- | --- | --- | --- | --- | --- | --- | --- | --- |
| All outcomes pooled | 17 | 0.53 | 0.18 – 0.87 | 84.24 | 76.08 – 89.62 | -0.78– 1.84 | .005^**^ | 5.66 |
| One effect size per study (lowest) | 17 | 0.50 | 0.16 – 0.84 | 83.48 | 74.79 – 89.18 | -0.80 – 1.80 | .007^**^ | 5.98 |
| One effect size per study (highest) | 17 | 0.56 | 0.20 – 0.91 | 84.24 | 76.08 – 89.61 | -0.79 – 1.90 | .004^**^ | 5.34 |
| Outliers removed | 15 | 0.39 | 0.24 – 0.54 | 50.66 | 10.76 – 72.71 | -0.02 – 0.81 | <.001^***^ | 7.89 |
| Influence analysis | 16 | 0.36 | 0.19 – 0.53 | 61.58 | 33.85 – 77.68 | -0.17 – 0.89 | <.001^***^ | 8.66 |
| Only low risk of bias | 4 | 0.41 | -0.16 – 0.98 | 64.99 | 0.00 – 88.12 | -0.67 – 1.49 | .107 | 7.50 |
| Three-level Model (CHE) | 21^a^ | 0.53 | 0.20 – 0.86 | 90.30 | - | -0.76 – 1.82 | .003^**^ | 5.64 |
| Publication bias corrections |  |  |  |  |  |  |  |  |
| Trim-and-fill method | 21 | 0.30 | -0.11 – 0.71 | 88.98 | 84.56 – 92.14 | -1.51 – 2.10 | .146 | 10.74 |
| Limit meta-analysis | 17 | -0.22 | -0.81 – 0.37 | 92.15 | - | -1.63 – 1.20 | .471 | 15.14 |
| Selection method | 17 | 0.08 | -1.09 – 1.24 | 95.79 | 86.72 – 99.25 | -2.00 – 2.15 | .898 | 45.66 |
| Subgroup analyses |  |  |  |  |  |  |  |  |
| Provision of IPD |  |  |  |  |  |  | .683 |  |
| Provided | 9 | 0.44 | 0.18 – 0.70 | 63.00 | 23.90 – 82.01 | -0.22 – 1.11 | .005^**^ | 6.90 |
| Did not provide | 8 | 0.59 | -0.21 – 1.39 | 91.17 | 85.01 – 94.80 | -1.70 – 2.88 | .127 | 5.00 |
| Type of intervention |  |  |  |  |  |  | .472 |  |
| CBT | 12 | 0.59 | 0.08 – 1.10 | 88.44 | 81.71 – 92.70 | -1.13 – 2.31 | .027^*^ | 4.98 |
| Non-CBT | 5 | 0.41 | 0.18 – 0.64 | 29.50 | 0.00 – 72.67 | 0.03 – 0.79 | .008^**^ | 7.46 |
| Type of control^b^ |  |  |  |  |  |  | .295 |  |
| CAU | 9 | 0.38 | 0.13 – 0.63 | 63.51 | 25.12 – 82.22 | -0.24 – 1.00 | .008^**^ | 8.20 |
| WL | 6 | 0.85 | -0.28 – 1.98 | 93.27 | 88.02 – 96.21 | -2.03 – 3.73 | .110 | 3.29 |
| Type of guidance |  |  |  |  |  |  | .666 |  |
| Guided | 9 | 0.45 | 0.13– 0.78 | 68.54 | 36.92 – 84.31 | -0.41 – 1.32 | .012^*^ | 6.71 |
| Self-guided | 8 | 0.61 | -0.16 – 1.37 | 90.80 | 84.28 – 94.62 | -1.57 – 2.78 | .104 | 4.83 |
| Diagnostic interview used? |  |  |  |  |  |  | .656 |  |
| Yes | 6 | 0.59 | 0.44 – 0.75 | 0.00 | 0.00 – 74.62 | 0.39 – 0.80 | <.001^***^ | 4.95 |
| No | 11 | 0.48 | -0.08 – 1.04 | 88.56 | 81.53 – 92.92 | -1.34 – 2.30 | .088 | 6.33 |
| Region |  |  |  |  |  |  | .648 |  |
| Western countries | 11 | 0.43 | 0.23 – 0.64 | 58.81 | 19.81 – 78.84 | -0.10 – 0.97 | <.001^***^ | 7.06 |
| Non-western countries | 6 | 0.65 | -0.54– 1.84 | 93.51 | 88.54 – 96.33 | -2.39 – 3.69 | .219 | 4.47 |
| *Note.* Abbreviations: 95%CI: 95% confidence Interval; 95%PI: 95% prediction interval; g: Hedges’ g; *I^2^*: I-square; k: number of studies; N: Number of observations; p: p-value; SE: Standard error.  ^a^ Four studies reported results of 2 measures of depressive symptoms.  ^b^ Two studies used other type of control than CAU or WL (namely, information leaflet or psychoeducation application).  ^*^ *p* < .05; ^**^ *p* < .01; ^***^ *p* < .001 | | | | | | | | |

## Conventional meta-analysis with sensitivity analyses

## Multivariate meta-regression analysis assessing the impact of IPD provision

|  | | Coef. | *95% CI* | *p* |
| --- | --- | --- | --- | --- |
| Providing vs non-providing IPD (dummy) |  | -0.14 | -1.19 – 0.91 | .768 |
| Risk of bias |  | 0.07 | -0.62 – 0.77 | .818 |
| Control group (dummy) |  | 0.52 | -0.69 – 1.72 | .364 |
| Western vs non-western country (dummy) |  | -0.22 | -1.31 – 0.86 | .656 |
| *Note.* Abbreviations: 95%CI: 95% confidence Interval; Coef.: coefficient; p: p-value.  ^*^ *p* < .05; ^**^ *p* < .01; ^***^ *p* < .001 | | | | |

## Forest plot

## Funnel plot

## Comparison of studies which did and did not provide individual participant data

|  | | **Provided (*k* = 9)** | **Did not provide (*k* = 8)** |  |
| --- | --- | --- | --- | --- |
|  | | ***M (SD)*** | ***M (SD)*** | ***p*** |
| **Sample size** |  | 109 (73) | 144 (115) | .455 |
|  |  | ***M (95% CI)*** | ***M (95% CI)*** |  |
| **Effect size** |  | 0.44 (0.18 – 0.70) | 0.59 (-0.21 – 1.39) | .683 |
|  |  | ***k (%)*** | ***k (%)*** |  |
| **Country** |  |  |  |  |
| Western |  | 6 (66.7%) | 5 (62.5%) | .999 |
| Non-western |  | 3 (33.3%) | 3 (37.5%) |  |
| **Intervention format** |  |  |  | .999 |
| Guided |  | 5 (55.6%) | 4 (50%) |  |
| Self-guided |  | 4 (44.4%) | 4 (50%) |  |
| **Control type** |  |  |  | .315 |
| Care as usual |  | 3 (42.9%) | 6 (75%) |  |
| Waitlist |  | 4 (57.1%) | 2 (25%) |  |
| **Recruitment** |  |  |  | .620 |
| Community |  | 7 (77.8%) | 5 (62.5%) |  |
| Screening |  | 2 (22.2%) | 3 (37.5%) |  |
| **Baseline severity** |  |  |  | .567 |
| Minimal |  | 2 (22.2%) | 1 (14.3%) |  |
| Mild |  | 1 (11.1%) | 3 (42.9%) |  |
| Moderate |  | 5 (55.6%) | 3 (42.9%) |  |
| Severe |  | 1 (11.1%) | 0 (0%) |  |
| **Risk of bias** |  |  |  | .399 |
| Low |  | 3 (33.3%) | 1 (12.5%) |  |
| Some concerns |  | 1 (11.1%) | 3 (37.5%) |  |
| High |  | 5 (55.6%) | 4 (50%) |  |
| *Note.* Studies which did and did not provide individual participant data were compared on continuous variables using the independent samples t-test, and on categorical variables by the Fisher’s exact test, because most cells had expected count of less than 5 (Field, 2013). The analyses were conducted in SPSS-Statistics, version 27.  Two studies that provided individual participant data, namely Sun and Zuccolo, used a different control group than care as usual or waitlist condition, and were therefore not included in the comparison of type of control.  To be able to compare studies using varying instruments on baseline severity, the baseline mean of the sample for each study was converted to the Beck Depression Inventory-II (Beck et al., 1996) based on established conversion methods (Furukawa et al., 2020; Leucht et al., 2018; Wahl et al., 2014). | | | | |

# **Appendix O. Participant Characteristics (Completers of Questionnaires Only)**

|  | **Valid *n*** | **Intervention**  ***n* = 551** | **Control**  ***n* = 543** | **Total**  ***n* = 1094** |
| --- | --- | --- | --- | --- |
|  | ***n* (%)** | ***n* (%)** | ***n* (%)** | ***n* (%)** |
| **Tertiary education** | 1091 | 420 (76.2) | 408 (75.56) | 828 (75.89) |
| **Full/part-time employment** | 805 | 269 (66.09) | 253 (63.57) | 522 (64.84) |
| **In a relationship** | 1087 | 512 (93.43) | 487 (90.35) | 999 (91.90) |
| **Ethnic minority** | 690 | 91 (26.22) | 83 (24.20) | 174 (25.22) |
| **Comorbid anxiety** | 1064 | 264 (49.25) | 257 (48.67) | 521 (48.97) |
| **Previous treatment** | 664 | 139 (41.37) | 128 (39.02) | 267 (40.21) |
|  | ***n* (%)** | ***M (SD)*** | ***M (SD)*** | ***M (SD)*** |
| **Age (years)** | 1091 | 31.47 (4.87) | 31.48 (5.00) | 31.48 (4.93) |
| **EPDS at baseline** | 782 | 12.76 (5.42) | 13.01 (6.30) | 12.89 (5.87) |
| **EPDS at post-treatment** | 595 | 9.84 (4.98) | 10.72 (5.83) | 10.30 (5.45) |
| **DASS – Anxiety subscale baseline** | 359 | 7.47 (6.29) | 6.99 (6.07) | 7.24 (6.18) |
| **DASS – Anxiety subscale post** | 325 | 3.15 (3.77) | 5.01 (4.86) | 4.11 (4.46) |
| **Number of children** | 564 | 1.40 (0.89) | 1.33 (0.75) | 1.37 (0.82) |
| *Note.* Abbreviations (alphabetical): EPDS: Edinburgh Postnatal Depression Scale; M: mean; n: number of participants; SD: standard deviation. | | | | |

# **Appendix P. Forest and funnel plots for the Individual Participant Data Meta-analysis**

## Forest plot

## Funnel plot

# **Appendix Q. The Strength of Evidence of Our Main Findings as Evaluated Based on the GRADE Assessment**

| **Analysis** | ***k*** | ***N*** | ***SMD*** | ***95% CI*** | **RoB** | **Inconsistency** | **Imprecision** | **Indirectness** | **Other considerations** | **Overall** |
| --- | --- | --- | --- | --- | --- | --- | --- | --- | --- | --- |
| Primary:  Reduction in depressive symptom severity (ITT) | 9 | 1094 | 0.30 | 0.03 – 0.55 | Not serious | Not serious | Not serious | Not serious | Serious | ⨁⨁⨁◯ Moderate |
| Reduction in depressive symptom severity (CC) | 9 | 875 | 0.30 | 0.04 – 0.55 | Not serious | Not serious | Not serious | Not serious | Not serious | ⨁⨁⨁⨁ High |
|  |  |  | ***RR*** |  |  |  |  |  |  |  |
| Response (ITT) | 9 | 1094 | 1.97 | 1.57 – 2.47 | Not serious | Serious | Not serious | Not serious | Not serious | ⨁⨁⨁◯ Moderate |
| Remission (ITT) | 9 | 1094 | 1.73 | 1.38 – 2.18 | Not serious | Serious | Not serious | Not serious | Serious | ⨁⨁◯◯ Low |

*Notes.* Abbreviations: 95%CI: 95% confidence Interval; CC: Complete-case analysis; ITT: Intention-to-treat analysis; k: number of studies; N: Number of observations; RoB: Risk of Bias; ß: Beta-coefficient.

We rated the items as described in previous research (Miguel et al., 2023):

**Risk of Bias:** Rated as not serious, because more than 60% of studies were low at risk of bias.

**Inconsistency:** When *I^2^* was over 75% and its 95% CI substentially overlapped with the category of considerable heterogeneity, meaning above 75%, measured by the median lying at 75% or higher, we rated this item as serious.

**Indirectness:** We rated all results as not serious on indirectness, because we only included studies in our meta-analysis if they directly compared the interventions of interest delivered to the participants of interest and measured the outcomes of interest.

**Imprecision:** As a rule of thumb, all analyses including more than 400 participants may be rated as not serious for imprecision.

**Other considerations:** In other considerations, we focused on publication bias. Egger’s test could not be calculated for any of the analyses, as we only included 9 studies. We therefore rated this item as serious whenever any analysis estimating treatment effects became non-significant as opposed to the main, significant, analysis.

**Overall score:** We considered high strength of evidence as a starting point and for every “serious” rating we then deducted one point, thus going from high through moderate to low and very low.

# **Appendix R. Sensitivity Analyses – Effectiveness of Internet-Based Interventions for Perinatal Depression**

|  | ***k*** | ***N*** | ***SMD [95%CI]***  ***(95% PI)*** | ***β-coefficient***  ***[95% CI]*** | ***Heterogeneity (SMD) [95% CI]*** | ***p*** |
| --- | --- | --- | --- | --- | --- | --- |
| **Influence Analysis** | 8 | 1051 | 0.21 [0.05 – 0.37]  (-0.12 – 0.53) | -2.11 [-3.71 - -0.51] | *I^2^* = 23.1  [0.0 – 64.7] | .017^*^ |
| **Only low risk of bias** | 5 | 620 | 0.34 [-0.26 – 0.94]  (-0.91 – 1.58) | -3.45 [-9.47 – 2.57] | *I^2^* = 73.3  [33.2 – 89.3] | .187 |
| **Three-level Model (CHE)** | 9 | 1094 | 0.29 [0.05 – 0.52]  (-0.25 – 0.82) | -2.91 [-5.29 - -0.53] |  | .023^*^ |
| **Main Effects: Complete case analysis** |  |  |  |  |  |  |
| Baseline depression severity | 9 | 875 |  | 4.19 [2.95 – 5.43] |  | <.001^***^ |
| Group |  |  | 0.30 [0.04 – 0.55]  (-0.27 – 0.86) | -2.98 [-5.54 - -0.41] | *I^2^* = 61.7  [20.8 – 81.5] | .028^*^ |
| **Main Effects: One-stage IPDMA** |  |  |  |  |  |  |
| Baseline depression severity | 9 | 1094 |  | 5.84 [5.11 – 6.57] | Intercept: *σ^2^* = 0.08 | <.001^***^ |
| Group |  |  | 0.30 [0.05 – 0.56]  (-0.47 – 1.08) | -3.03 [-5.59 - -0.46] | *τ^2^* = 0.10 | .021^*^ |
| **Main Effects: Response** |  |  | ***RR [95%CI]***  ***(95% PI)*** |  |  |  |
| Baseline depression severity | 9 | 1094 |  | 0.14 [-0.08 – 0.35] |  | .175 |
| Group |  |  | 1.97 [1.57 – 2.47]  (1.00 – 3.88) |  | *I^2^* = 78.9  [60.4 – 88.8] | <.001^***^ |
| **Main Effects: Remission** |  |  |  |  |  |  |
| Baseline depression severity | 9 | 1094 |  | -0.79 [-1.15 - -0.44] |  | <.001^***^ |
| Group |  |  | 1.73 [1.38 – 2.18]  (0.85 – 3.51) |  | *I^2^* = 91.8  [86.7 – 94.9] | <.001^***^ |
| **Publication bias corrections (group)** |  |  | ***SMD [95%CI]***  ***(95% PI)*** |  |  |  |
| Trim-and-fill method | 11 |  | 0.20 [0.12 – 0.51]  (-0.66 – 1.05) | -1.97 [-5.12 – 1.19] | *I^2^* = 68.7  [41.5 – 83.3] | .196 |
| Limit meta-analysis | 9 | 1094 | 0.12 [-0.20 – 0.43]  (-0.52 – 0.75) | -1.15 [-4.32– 2.03] |  | .480 |
| Selection method | 9 | 1094 | 0.43 [0.04 – 0.81]  (-0.16 – 1.01) | -4.26 [-8.11 - -0.42] | *I^2^* = 62.1  [0.0 – 90.3] | .030^*^ |
| **Subgroup analyses (group)** |  |  |  |  |  |  |
| **Guidance** |  |  |  |  |  | .083 |
| Guided interventions | 5 | 277 | 0.51 [-0.01 – 1.02]  (-0.43 – 1.45) | -5.06 [-10.23 – 0.11] | *I^2^* = 51.0  [0.0 – 82.0] | .053 |
| Self-guided interventions | 4 | 817 | 0.15 [-0.12 – 0.42]  (-0.33 – 0.63) | -1.50 [-4.24 – 1.23] | *I^2^* = 42.2  [0.0 – 80.6] | .178 |
| **Control conditions** |  |  |  |  |  | .096 |
| Care as usual | 3 | 311 | 0.67 [-0.46 – 1.80]  (-1.34 – 2.68) | -6.68 [-18.00– 4.64] | *I^2^* = 69.8  [0.0 – 91.2] | .126 |
| Waitlist condition | 4 | 351 | 0.19 [-0.19 – 0.57]  (-0.55 – 0.92) | -1.88 [-5.66 – 1.90] | *I^2^* = 41.4  [0.0 – 80.3] | .212 |
| **Diagnostic interview** |  |  |  |  |  | .231 |
| Yes | 4 | 227 | 0.53 [-0.29 – 1.34]  (-1.00 – 2.05) | -5.25 [-13.35 – 2.85] | *I^2^* = 63.2  [0.0 – 87.6] | .131 |
| No | 5 | 867 | 0.20 [-0.05 – 0.45]  (-0.27 – 0.67) | -2.01 [-4.52 – 0.50] | *I^2^* = 50.5  [0.0 – 81.8] | .090 |
| **Sensitivity analyses (group)** |  |  |  |  |  |  |
| Only interventions delivered postnatally ^a^ | 6 | 693 | 0.36 [-0.10 – 0.83]  (-0.67 – 1.40) | -3.62 [-8.28 – 1.03] | *I^2^* = 71.8  [34.6 – 87.8] | .102 |
| **Meta-regression analyses (group)** |  |  |  |  |  |  |
| Risk of bias | 9 | 1094 |  | 0.12 [-5.64 – 5.88] |  | .962 |
| Number of sessions | 9 | 1094 |  | -0.03 [-1.88 – 1.82] |  | .974 |
| *Notes.* Abbreviations: 95%CI: 95% confidence Interval; k: number of studies; N: Number of observations; p: p-value; RR: relative risk.  ^*^ *p*-value < .05; ^**^ *p*-value < .01; ^***^ *p*-value < .001  ^a^ – It was not possible to assess only interventions delivered antenatally (*k* = 2). | | | | | | |

# **Appendix S. Sensitivity Analyses and Additional Information for the Adherence Analysis**

## Prognostic Factors of Adherence to Internet-Based Interventions for Perinatal Depression Analyzed with Poisson Regression

|  | ***k*** | ***N*** | ***β-coefficient [95% CI] (95% PI)*** | ***I^2^* [95% CI]** | ***p*** |
| --- | --- | --- | --- | --- | --- |
| Baseline depressive symptoms | 7 | 313 | -0.04 [-0.14 – 0.05] (-0.23 – 0.14) | 0.0 [0.0 – 70.8] | .296 |
| Age (continuous) | 7 | 310 | 0.04 [-0.02 – 0.11] (-0.14 – 0.23) | 0.0 [0.0 – 70.8] | .138 |
| Education (tertiary vs lower) | 7 | 313 | -0.17 [-0.37 – 0.02] (-0.70 – 0.36) | 0.0 [0.0 – 70.8] | .076 |
| Relationship status (no vs yes) | 4 | 161 | 0.00 [-0.31 – 0.31] (-1.47 – 1.47) | 0.0 [0.0 – 84.7] | .999 |
| Employment status (employed vs other) | 5 | 271 | 0.03 [-0.09 – 0.16] (-0.61 – 0.68) | 0.0 [0.0 – 79.2] | .485 |
| Baseline anxiety symptoms (continuous) | 6 | 273 | 0.02 [-0.15 – 0.20] (-0.23 – 0.28) | 0.0 [0.0 – 74.6] | .745 |
| Comorbid anxiety (no vs yes) | 7 | 299 | 0.02 [-0.27 – 0.30] (-0.42 – 0.45) | 0.0 [0.0 – 70.8] | .894 |
| Previous treatment (no vs yes) | 5 | 198 | 0.04 [-0.13 – 0.21] (-0.48 – 0.56) | 0.0 [0.0 – 79.2] | .562 |
| Number of children (continuous) | 4 | 180 | -0.07 [-0.20 – 0.06] (-0.53 – 0.39) | 0.0 [0.0 – 84.7] | .201 |
| Minority status (no vs yes) | 3 | 80 | 0.11 [-0.25 – 0.47] (-2.02 – 2.23) | 0.0 [0.0 – 89.6] | .324 |
| *Notes.* Abbreviations: 95%CI: 95% confidence Interval; 95% PI: 95% prediction interval; k: number of studies; N: Number of observations; p: p-value; SE: Standard error.  ^*^ *p*-value < .05; ^**^ *p*-value < .01; ^***^ *p*-value < .001 | | | | | |

## Prognostic Factors of Adherence to Internet-Based Interventions for Perinatal Depression Analyzed Separately for Guided Interventions (Beta Regression)

|  | ***k*** | ***N*** | ***β-coefficient [95% CI] (95%PI)*** | ***I^2^ [95% CI]*** | ***p*** |
| --- | --- | --- | --- | --- | --- |
| Baseline depressive symptoms | 5 | 131 | -0.18 [-0.59 – 0.22] (-0.87 – 0.50) | 39.8 [0.0 – 77.8] | .281 |
| Age (continuous) | 5 | 128 | 0.09 [-0.28 – 0.46] (-0.53 – 0.71) | 32.8 [0.0 – 74.5] | .539 |
| Education (tertiary vs lower) | 4 | 115 | -0.18 [-0.98 – 0.63] (-2.39 – 3.74) | 0.0 [0.0 – 89.6] | .535 |
| Relationship status (no vs yes) | 3 | 63 | 0.67 [-1.93 – 3.27] (-1.34 – 0.98) | 0.0 [0.0 – 84.7] | .382 |
| Baseline anxiety symptoms (continuous) | 3 | 86 | 0.08 [-0.68 – 0.83] (-0.97 – 1.12) | 22.5 [0.0 – 91.9] | .703 |
| Comorbid anxiety (no vs yes) | 5 | 128 | -0.24 [-0.80– 0.32] (-0.88 – 0.39) | 0.0 [0.0 – 79.2] | .293 |
| Previous treatment (no vs yes) | 4 | 100 | -0.19 [-0.63 – 0.25] (-0.98 – 0.60) | 0.0 [0.0 – 84.7] | .266 |
| *Notes.* Abbreviations: 95%CI: 95% confidence Interval; 95%PI: 95% prediction interval; k: number of studies; N: Number of observations; p: p-value; SE: Standard error.  ^*^ *p*-value < .05; ^**^ *p*-value < .01; ^***^ *p*-value < .001 | | | | | |

# **References (Supplementary materials)**

Beck, A. T., Steer, R. A., & Brown, G. K. (1996). Beck depression inventory.

Borenstein, M., Hedges, L. V., Higgins, J. P., & Rothstein, H. R. (2021). *Introduction to meta-analysis*. John Wiley & Sons.

Borenstein, M., Higgins, J. P., Hedges, L. V., & Rothstein, H. R. (2017). Basics of meta‐analysis: I2 is not an absolute measure of heterogeneity. *Research synthesis methods*, *8*(1), 5-18.

Carpenter, J. R., & Smuk, M. (2021). Missing data: A statistical framework for practice. *Biometrical Journal*, *63*(5), 915-947.

Cheung, M. W.-L. (2014). Modeling dependent effect sizes with three-level meta-analyses: a structural equation modeling approach. *Psychological methods*, *19*(2), 211.

Cox, J., & Holden, J. (2003). *Perinatal mental health: A guide to the edinburgh postnatal depression scale (EPDS)*. Royal College of Psychiatrists.

Field, A. (2013). *Discovering statistics using IBM SPSS statistics*. sage.

Firth, D. (1993). Bias reduction of maximum likelihood estimates. *Biometrika*, *80*(1), 27-38.

Furukawa, T. A., Reijnders, M., Kishimoto, S., Sakata, M., DeRubeis, R. J., Dimidjian, S., Dozois, D. J., Hegerl, U., Hollon, S. D., & Jarrett, R. B. (2020). Translating the BDI and BDI-II into the HAMD and vice versa with equipercentile linking. *Epidemiology and psychiatric sciences*, *29*, e24.

Groenwold, R. H., Moons, K. G., Peelen, L. M., Knol, M. J., & Hoes, A. W. (2011). Reporting of treatment effects from randomized trials: a plea for multivariable risk ratios. *Contemporary clinical trials*, *32*(3), 399-402.

Hanson, V. D. (2007). *Carnage and Culture: Landmark Battles in the Rise to Western Power*. Anchor.

Harrer, M., Cuijpers, P., Schuurmans, L. K., Kaiser, T., Buntrock, C., van Straten, A., & Ebert, D. (2023). Evaluation of randomized controlled trials: a primer and tutorial for mental health researchers. *Trials*, *24*(1), 562.

Hedges, L. V., & Olkin, I. (2014). *Statistical methods for meta-analysis*. Academic press.

Higgins, J. P., Thompson, S. G., Deeks, J. J., & Altman, D. G. (2003). Measuring inconsistency in meta-analyses. *bmj*, *327*(7414), 557-560.

IntHout, J., Ioannidis, J. P., & Borm, G. F. (2014). The Hartung-Knapp-Sidik-Jonkman method for random effects meta-analysis is straightforward and considerably outperforms the standard DerSimonian-Laird method. *BMC medical research methodology*, *14*, 1-12.

Jiang, H., Kulkarni, P. M., Mallinckrodt, C. H., Shurzinske, L., Molenberghs, G., & Lipkovich, I. (2017). Covariate adjustment for logistic regression analysis of binary clinical trial data. *Statistics in Biopharmaceutical Research*, *9*(1), 126-134.

Karyotaki, E., Kemmeren, L., Riper, H., Twisk, J., Hoogendoorn, A., Kleiboer, A., Mira, A., Mackinnon, A., Meyer, B., & Botella, C. (2018). Is self-guided internet-based cognitive behavioural therapy (iCBT) harmful? An individual participant data meta-analysis. *Psychological Medicine*, *48*(15), 2456-2466.

Kernot, J., Olds, T., Lewis, L. K., & Maher, C. (2015). Test-retest reliability of the English version of the Edinburgh Postnatal Depression Scale. *Archives of women's mental health*, *18*, 255-257.

Knudsen, D. C. (1992). Generalizing poisson regression: Including apriori information using the method of offsets. *The Professional Geographer*, *44*(2), 202-208.

Kroenke, K., Spitzer, R. L., & Williams, J. B. (2001). The PHQ‐9: validity of a brief depression severity measure. *Journal of general internal medicine*, *16*(9), 606-613.

Leucht, S., Fennema, H., Engel, R. R., Kaspers-Janssen, M., & Szegedi, A. (2018). Translating the HAM-D into the MADRS and vice versa with equipercentile linking. *Journal of affective disorders*, *226*, 326-331.

Miguel, C., Amarnath, A., Akhtar, A., Malik, A., Baranyi, G., Barbui, C., Karyotaki, E., & Cuijpers, P. (2023). Universal, selective and indicated interventions for supporting mental health at the workplace: an umbrella review of meta-analyses. *Occupational and environmental medicine*, *80*(4), 225-236.

Morris, T. P., Walker, A. S., Williamson, E. J., & White, I. R. (2022). Planning a method for covariate adjustment in individually randomised trials: a practical guide. *Trials*, *23*(1), 328.

Morris, T. P., White, I. R., & Royston, P. (2014). Tuning multiple imputation by predictive mean matching and local residual draws. *BMC medical research methodology*, *14*, 1-13.

Naimi, A. I., Cole, S. R., & Kennedy, E. H. (2017). An introduction to g methods. *International journal of epidemiology*, *46*(2), 756-762.

Pustejovsky, J. E., & Tipton, E. (2022). Meta-analysis with robust variance estimation: Expanding the range of working models. *Prevention Science*, *23*(3), 425-438.

Riley, R. D., Ensor, J., Hattle, M., Papadimitropoulou, K., & Morris, T. P. (2023). Two‐stage or not two‐stage? That is the question for IPD meta‐analysis projects. *Research synthesis methods*, *14*(6), 903-910.

Robins, J. (1986). A new approach to causal inference in mortality studies with a sustained exposure period—application to control of the healthy worker survivor effect. *Mathematical modelling*, *7*(9-12), 1393-1512.

Tong, L., Panagiotopoulou, O., Miguel, C., Harrer, M., Amarnath, A., Ciharova, M., Gemmill, A., Salamanca-Sanabria, A., Williams, A., Fonseca, A., Zarski, A., Geraedts, A., van Straten, A., Raevuori, A., Arntz, A., Ghosh, A., Meyer, B., Allenhof, C., Lukas, C., . . . Karyotaki, E. (2026). Predictors of Treatment Adherence in Internet-based Interventions for Depression: An Individual Participant Data Meta-Analysis. *Nature Mental Health*, 1-11. https://doi.org/https://doi.org/10.1038/s44220-026-00707-4

Van Buuren, S. (2012). *Flexible imputation of missing data* (Vol. 10). CRC press Boca Raton, FL.

Vansteelandt, S., & Keiding, N. (2011). Invited commentary: G-computation–lost in translation? *American journal of epidemiology*, *173*(7), 739-742.

Viechtbauer, W., & Cheung, M. W. L. (2010). Outlier and influence diagnostics for meta‐analysis. *Research synthesis methods*, *1*(2), 112-125.

Wahl, I., Löwe, B., Bjorner, J. B., Fischer, F., Langs, G., Voderholzer, U., Aita, S. A., Bergemann, N., Brähler, E., & Rose, M. (2014). Standardization of depression measurement: a common metric was developed for 11 self-report depression measures. *Journal of clinical epidemiology*, *67*(1), 73-86.

Wang, L., Kroenke, K., Stump, T. E., & Monahan, P. O. (2021). Screening for perinatal depression with the Patient Health Questionnaire depression scale (PHQ-9): A systematic review and meta-analysis. *General hospital psychiatry*, *68*, 74-82.

Williamson, T., Eliasziw, M., & Fick, G. H. (2013). Log-binomial models: exploring failed convergence. *Emerging themes in epidemiology*, *10*, 1-10.
